# Metal/semiconductor interfaces in nanoscale objects: synthesis, emerging properties and applications of hybrid nanostructures

**DOI:** 10.1039/c9na00729f

**Published:** 2020-03-02

**Authors:** Michael Volokh, Taleb Mokari

**Affiliations:** Department of Chemistry, Ilse Katz Institute for Nanoscale Science and Technology, Ben-Gurion University of the Negev Beer-Sheva 8410501 Israel mokari@bgu.ac.il

## Abstract

Hybrid nanostructures, composed of multi-component crystals of various shapes, sizes and compositions are much sought-after functional materials. Pairing the ability to tune each material separately and controllably combine two (or more) domains with defined spatial orientation results in new properties. In this review, we discuss the various synthetic mechanisms for the formation of hybrid nanostructures of various complexities containing at least one metal/semiconductor interface, with a focus on colloidal chemistry. Different synthetic approaches, alongside the underlying kinetic and thermodynamic principles are discussed, and future advancement prospects are evaluated. Furthermore, the proved unique properties are reviewed with emphasis on the connection between the synthetic method and the resulting physical, chemical and optical properties with applications in fields such as photocatalysis.

## Abstract 2

Multi-component crystals of various shapes, sizes and compositions (hybrid nanostructures) are much sought-after functional materials. Herein, the synthesis and emerging properties of nanomaterials with metal/semiconductor interface(s) are reviewed.

## Graphical abstract

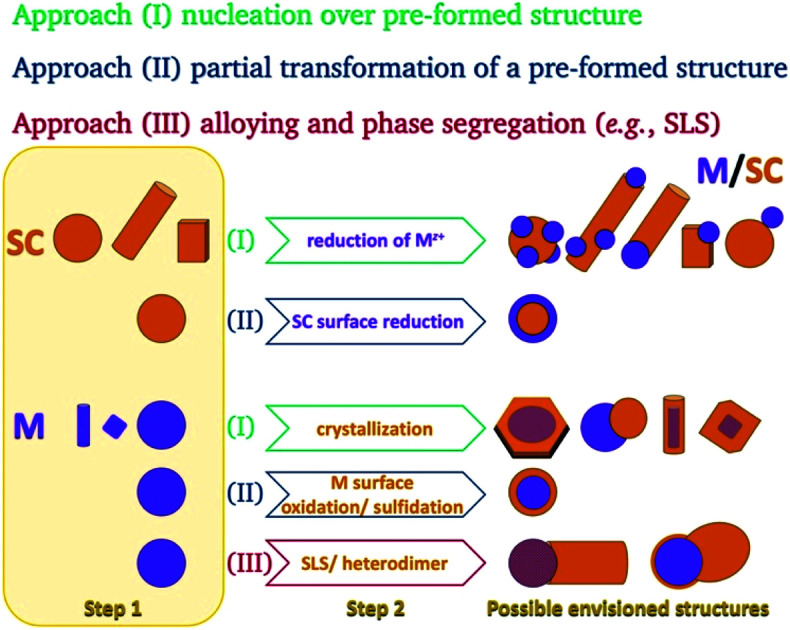

## 1. Introduction

Today, different nano-sized objects are synthesized, manipulated, characterized, and utilized in a plethora of scientific and technological applications. Every B.Sc. student in chemistry, physics, and most branches of engineering has heard of ‘nano’ objects such as carbon nanotubes or quantum dots (QD). Colloidal QDs are considered a masterpiece of modern nanoscience. The development of the hot-injection method^1^ in 1993 allowed studying the unique optical properties of QDs, since the surfactant-assisted precision synthetic method gives controlled surface chemistry with narrow size distributions and uniform morphologies.^2^ The QD, which is a quantum-confined semiconductor nanocrystal, is just one excellent example of the rapid progress of this relatively new field of science, from the theoretical description of quantum-confinement, which explains the unique photophysical properties, to other calculated physical properties,^3^ through different applications in electronic and optoelectronic devices,^4^ to commercialization in displays and bio-labeling, which are already on the market.^5^

With available pathways for the controlled synthesis of various shapes, sizes, and compositions of nanoscale objects (see for example reviews by van Embden *et al.*^6^ and Hyeon and co-workers^7–9^ on various colloidal synthetic methods), scientists looked for ways to combine two (or more) nano-objects into hybrid nanostructures (HNS) and potentially obtain materials with new properties. Continuing with the QD example, varying the deposition of a second semiconductor crystal on the existing QD results in a hybrid semiconductor/semiconductor (SC/SC) interface; some synthesized examples include core/(multi)shell,^10–18^ yolk/shell^19–21^ and heterodimers (usually two or more quasi-spherical shapes forming a heterojunction^22–24^ as well as acorn-style structures^25^). When the QD serves as a seed for an anisotropic structure such as a rod,^26^ tetrapod^26,27^ or octapod,^28^ more complex structures result,^29^ where the seed (junction) and arm are different SCs. Obviously, if the starting material is anisotropic (*e.g.*, nano-rod, -wire, -belt, -ribbon, -sheet, -pyramid)^6,30,31^ or even branched,^32^ other hybrid SC/SC nanostructures (possibly with mixed dimensionalities) result—ranging from simple heterostructures through rod-in-rod, dumbbells, dot-in-rod and graded shells,^33^ to ‘double’ QD fixed in a rod with unique optoelectronic properties.^34^ The examples thus far focused on the SC/SC interface, but a vast body of knowledge has been acquired on interfacing a SC with a metal (M)—these hybrid nanostructures (HNSs) are the topic of this review—and we will begin with discussion of binary systems in Section 2. For detailed analysis of the electronic coupling between a semiconductor, a metal and variable (insulating) interfaces, we refer the reader to the thorough review by Vilan and Cahen.^35^

Both shape and size dictate the electronic structure in metals^36^ and in semiconductors due to different quantum-confinement effects.^37^ Noteworthy, many electronic and optical properties of nanocrystals result not only from dimensionality and size but also from surface properties.^38^ Formation of an HNS could result in a mere combination of the individual material's properties (fluorescence, magnetism, plasmonic response, catalytic activity and so forth). However, usually, new properties arise from both the modifications at the surface of the original constituents and the newly formed interface, ranging from inherent physical properties to functional applications such as controlled self-assembly into superlattices without the inherent anisotropy of the HNS.^39^ The main criterion of distinction between the various possible nanohybrids is the type of the interface present: SC/SC,^16^ M/M,^40^ and M/SC.^41,42^ For practical applications such as photocatalysis, multiple interfaces can also be combined.^43^

In this review, we focus on the tremendous synthetic progress achieved in the formation of hybrid inorganic M/SC nanostructures and their interfaces, mainly in colloidal liquid-phase syntheses. The building blocks of HNSs are commonly referred to as nanocrystals (NCs) and nanoparticles (NPs), with NCs usually referring to a semiconductor and NPs—despite being a general term—usually implies a metallic nano-object. Since the chemical principles of nanoscale synthesis have been reviewed,^44^ we will emphasize the mechanisms of growth and precise control over the formed interfaces between metals and semiconductors (with a special focus on the II–VI family—the metal-chalcogenide (O, S, Se, Te) materials and formation of anisotropy or asymmetry). We refer the reader to excellent reviews by Cozzoli and co-workers,^45,46^ Manna and co-workers,^47^ and Banin and co-workers,^48^ who set the groundwork for the description of hybrid nanostructures, and a recent review from Zhang and co-workers on epitaxial multicomponent HNSs.^49^ These studies give a broad overview of the development of the field and various possible interfaces that can form alongside thermodynamic requirements for the growth of HNSs. Having this knowledge at the reader's disposal allows us to select illustrative HNS examples (including knowledge accumulated in our group) to elaborate the chemical principles of colloidal metal–semiconductor HNS synthesis, and explore new synthetic approaches for the formation of HNSs with increasing complexity (Section 3), which were developed during the last decade such as using a total-synthesis framework for formation of high-order hybrid nanoparticles, developed by Schaak and co-workers.^50,51^

Furthermore, we mention in Section 2.2 additional non-semiconductor transition metal–nonmetal compounds, which have many synthetic similarities to semiconductors and can form during HNS synthesis. Finally, we demonstrate how the new emergent properties of the various HNSs are beneficial in selected applications, such as (photo)catalysis (Section 4).

## 2. Formation of binary metal/semiconductor hybrid nanostructures

Discussing M/SC interfaces raises several interesting scientific questions. What is the mechanism behind the possibility of forming an intimate interface between two different (ordered) materials? What keeps the final structure stable? What are the underlying thermodynamic, kinetic and mechanistic principles (and details) that are responsible for the intermediate steps of the reaction? What is the interplay between the chosen precursors and the required external energy source (photons, electrons, thermal energy, electrochemical potential, *etc.*) required to drive the reaction forward? What are the effects of the size and shape of the domains and the interface on the possible structures? And finally, which new phenomena arise from the successful formation of the hybrid at the nanoscale?

With these scientific questions in mind, a simplified M/SC HNS application-oriented design scheme may be proposed: (i) determine the relevant properties of the individual metal and semiconductor domains; (ii) specify the physical, mechanical, chemical, optical or electronic properties that are expected to change or remain unaltered after the hybrid structure has been synthesized; (iii) verify that the property-combination is suitable for the potential applications. Once decided, the most-suitable synthetic procedure can be designed. Our goal in this review is to give an overview of the most up-to-date answers to the chemistry-of-materials questions, with a focus on the synthetic possibilities.

It is impossible to provide an adequate classification of all possible property combinations, yet a useful generalization would be to classify the metal and semiconductor roles. The intrinsic role of a metal (or a bimetallic compound) can be classified, as common for metal NPs,^52^ into plasmonic (*e.g.*, Cu, Ag, Au), magnetic (Fe, Ni, Co), and catalytic properties (*e.g.*, Pt-group metals). To these roles, we can add the electronic properties of the metal, which are intimately connected to the semiconductor—specifically, electrical conductivity and Fermi level (*E*_F_). In the case of the semiconductor, the most important property is usually the bandgap (*E*_g_), which defines the material and its optical absorption, though the entire electronic structure (*i.e.*, density of states) is important for planning an applicable M/SC interface: Fermi level, doping, band positions and expected defect levels, carrier diffusion length, fluorescence yield and even plasmonic response due to defects in semiconductors (*e.g.*, vacancies in copper sulfide and impurity doping in metal oxides).^53^

### 2.1 Colloidal routes to form M/SC interfaces

In the colloidal synthesis realm, nanocrystals either crystallize on predetermined sites or form in a solution due to a nucleation event. Since homogeneous nucleation is less favorable than heterogeneous nucleation on a pre-existing nucleation site, the planned placement of nucleation sites allows the synthesis of complicated nanostructures in a variety of techniques. The same considerations apply during the growth of a material—the free energy barrier is lower for the nucleation of a precursor on an existing crystal than for spontaneous homogeneous nucleation.

**Fig. 1:**
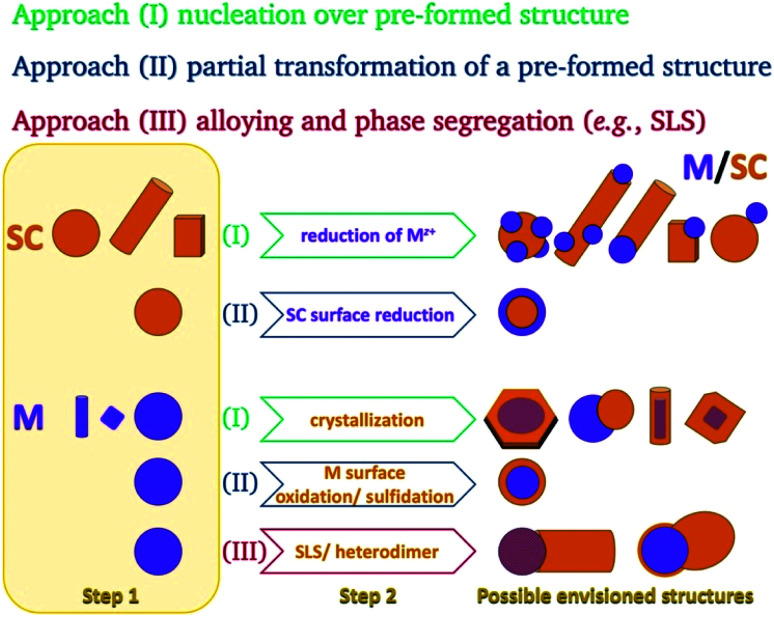
Schematic options to form nanoscale colloidal-phase heterostructures using a starting semiconductor (SC, orange) or metal (M, purple) structure. Approach (I): nucleation of M over SC or SC over M; approach (II): partial transformation of the first structure (reduction at the surface of a binary SC's cation to the metal or surface transformation of a metal into a semiconductor (*e.g.*, oxidation or sulfidation)); approach (III): precipitation out of a metal-alloy core, which either forms a heterodimer or a nanorod *via* a solid–liquid–solid (SLS) mechanism, where the SC precursors are dissolved in the metal tip (alloying) and precipitate out of it, resulting in the formation of an M/SC interface.

This basic thermodynamic principle,^54^ alongside kinetic control over the number of available nucleation sites and precursors, allows interfacing two materials. Fig. 1 schematically depicts the conventional two-step approaches for the formation of SC/M and M/SC interfaces. One can divide the syntheses according to the first material employed: semiconductor or metal. When the first material is a semiconductor, the subsequent metal domain formation is usually performed by reducing a metal cation—either using a photo-assisted process (Section 2.1.1) or chemically (Section 2.1.2). This is approach (I), where a reduction of metal from the solution results in nucleation and growth on the SC. The other option, approach (II), is a partial chemical reduction of the SC at the surface of the nanocrystal, resulting in metal coating or domains. This is a relatively rare synthetic approach with a starting SC (*e.g.*, Cu_2_O into Cu_2_O/Cu^0^).^55^

The colloidal approaches for the synthesis of metal NPs (as the anchor for a subsequent SC growth) are dominated by chemical reduction both in aqueous and organic-solvent environments. The most prominent example is the citrate-assisted reduction of gold from Au(iii) to Au^0^, but alcohols, amines,^56^*etc.* are also common reducing agents. Noticeable alternatives include the use of metal carbonyl complexes, where the metal's oxidation state is 0.^57^ To stabilize the resulting metallic NPs, the typical surfactants are long-chain organic molecules, polymers (*e.g.*, PVP), and micelles (formed using hexadecyl-trimethyl-ammonium bromide, CTAB, in an aqueous environment for example).

The metal can serve as the seed for growth of SC crystals (approach (I), discussed in Section 2.1.3), as the substrate for chemical transformations (approach (II), *e.g.*, surface oxidation and sulfidation, discussed in Section 2.1.4), or as the catalyst for a solution–liquid–solid (SLS) growth (approach (III), Section 2.1.5).

#### 2.1.1 Photodeposition of metals on semiconductor nanostructures

**Fig. 2:**
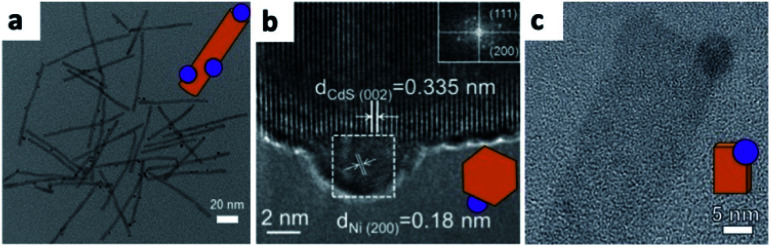
Examples of metal photodeposition on SCs. (a) Pt/CdSe–CdS NRs *via* a non-selective Pt domain photodeposition reaction carried out in an ionic liquid, adapted from ref. 61, Creative Commons Attribution 4.0 International Public License, published by Beilstein-Institut, copyright 2019. (b) Ni/CdS NCs, adapted with permission from ref. 67, American Chemical Society, copyright 2016. (c) Ni/CdS nanosheets, adapted with permission from ref. 68, American Chemical Society, copyright 2015.

When using a SC nanostructure, one can nucleate a metal crystal on its surface by reduction. The reduction can be induced either chemically (Section 2.1.2) or photochemically (this section). Illuminating a semiconductor and using the excited electrons to reduce an existing metal-containing molecule in the surrounding solution is a photodeposition method. The Weller group deposited silver on ZnO nanorods (NRs) in an ethanediol/water (2 : 1 v/v) mixture.^58^ Alivisatos and co-workers have shown photodeposition of Pt on CdS and CdSe–CdS core–shell NRs in toluene.^59^ The control over the number of Pt NPs per rod (a wide distribution in the 0–6 range) proved to be hard, with the best control achieved on well-passivated CdSe–CdS NRs, where most NRs contained a single Pt domain, close to the CdSe core. Others have deposited different metals on various semiconductors of diverse shapes.^60^ Recently, photodeposition in ionic liquids has been carried out by Hill and co-workers, who have shown non-selective deposition of Pt, Au and Ag on CdSe–CdS NRs with similar results to the common amine-capped colloidal synthesis in toluene (Fig. 2a).^61^

Carbone *et al.* have used UV light in 9 : 1 v/v CHCl_3_ : ethanol (EtOH) to achieve controlled reduction of gold on the tips of CdS and CdSe(core)@(shell)CdS NRs.^62^ The advantage of this method is that the EtOH acts as an electron donor, reacting with the photogenerated holes in the semiconductor, thus allowing the photogenerated electron in the rod to reduce a gold cation. This method allowed the synthesis of large Au domains on CdSe@CdS or CdS NR tips and nano-dumbbells. The fact that the large domain formation is selective at one tip is a significant synthetic achievement since photodeposition is not usually selective. By measuring the absorption peak location stemming from the metal domain's plasmonic response, transmission electron microscopy (TEM) analysis and controlled addition of a gold precursor, the authors deduce that the mechanism is mainly through selective drift of the photogenerated electrons towards the gold tip (forming, at first, alongside other Au domains), which enhances further reduction of the large metal tip, acting as an electron sink.

Fernando *et al.* have shown that gold readily photodeposits on ZnO at high-energy sites as facet edges and corners when the capping ligand is a labile amine, but when a more strongly bound dodecanethiol (DDT) is used, the deposition is quenched.^63^ Furthermore, since the deposition rate depends on the interfacial electron transfer from the ZnO to the cationic gold complex, changing the solvent could determine whether multiple small Au domains are formed, or fewer larger Au clusters result (when the reaction conditions favor charge accumulation).^63^ The main drawback of the photodeposition approach discussed thus far is its non-selective nature. This work also demonstrates another limitation, which is the non-epitaxial nature of the formed heterostructure.^63^ In a successive study, it was demonstrated that a sequential photodeposition of Pt over photodeposited Au/ZnO HNS results in a non-selective Pt deposition with Pt and Au domains distributed over the ZnO core, while changing the procedure to Pt photodeposition on an Au-seeded/ZnO HNS (the focus of Section 2.1.3) allows selective deposition of Pt on the existing epitaxial Au domains/ZnO.^64^

In aqueous solutions, when TiO_2_ NPs are illuminated using UV radiation in the presence of KMnO_4_, the reduction results in the formation of a TiO_2_/MnO_2_ HNS with manganese oxide domains. As most domains are present on one side, a Janus particle is formed, which can catalytically decompose H_2_O_2_ as a proof-of-concept ‘nanomotor’.^65^ Another example is a CdS–nickel oxide hybrid, which consists mostly of Ni_2_O_3_, which is photodeposited using UV irradiation in an aqueous environment containing Ni^2+^ salt and nitrites.^66^ These two examples demonstrate that metal-oxides and -hydroxides are easily formed in aqueous environments.

Partial oxidation of Ni in CdS/Ni HNSs due to the presence of water was suggested by Chai *et al.* to be responsible for less efficient photocatalytic dehydrogenation of alcohols—for this reason, they performed the Ni photodeposition in methanol (Fig. 2b).^67^ Simon *et al.*, managed to synthesize multiple decorating elemental Ni NPs on a cysteine-stabilized aqueous colloid of CdS NRs by photodeposition with low amounts of NiO and Ni(OH)_2_.^69^ They ascribe this success to surface-trapped photoexcited electrons, which reduce the nickel cations in their vicinity; once a metallic cluster is formed, it serves as an electron sink to further reduce and form Ni NPs with diameters of about 5 nm (though 2 nm NPs were also detected). Kuno and co-workers selectively deposited Ni on 2D CdS nanosheets in an Ar-purged 10% EtOH aqueous solution under 405 nm laser illumination (Fig. 2c).^68^ Mahler and co-workers used photo-assisted reduction to form large Au domains on 2D CdSe/CdZnS.^70^

Another innovative use of photoexcitation in HNSs is the transformation of a CdS NR/Pt tip into copper-deficient Cu_2_S NR/Pt using light-induced cation exchange in an aqueous environment as demonstrated by Manzi *et al.* for CO_2_ reduction applications.^71^

#### 2.1.2 Chemical reduction of metals on semiconductor nanostructures

Deposition of gold on a single side or on both sides of CdSe NCs was first demonstrated by the reduction of gold(iii) chloride at room temperature (rt) in a colloidal suspension of CdSe nanostructures (QDs, NRs and tetrapods) in toluene.^72–74^ In this procedure, dodecylamine (DDA) acts both as the surfactant of the CdSe nanostructure and as the reducing agent, while didodecyldimethylammonium bromide (DDAB) is the surfactant for the gold in the organic medium.

**Fig. 3:**
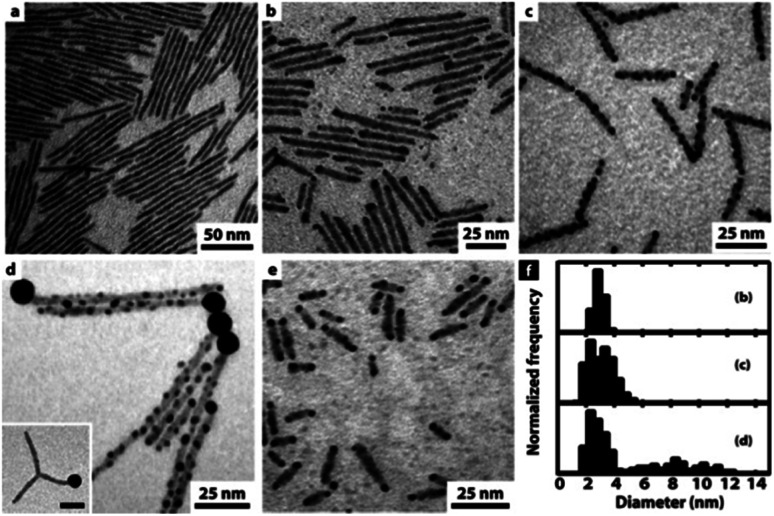
Gold decoration of CdS NRs (average dimensions 3.9 × 100 nm) with and without exposure to air. (a) Deaerated synthesis results in no gold deposition; (b) at the initial stage (∼30 min), gold nucleates preferentially on the NRs' tips; (c) after 150 min, considerable gold growth on defect sites along the NRs; (d) after long reaction times (3 days), large gold domains are formed *via* a ripening mechanism, *i.e.*, large domains are formed at the expense of small NPs, which have a higher tendency to dissolute. (e) Gold nucleation on CdS NRs with a smaller aspect ratio (3.9 × 20 nm) after *ca.* 80 min. (f) The corresponding histograms of Au domain diameters: size evolution over time for images (b–d). Reproduced from ref. 75 with permission from the American Chemical Society, copyright 2006.

Further investigation of a similar system of CdS NRs showed an interesting strategy to control the amount and location of the gold NPs—the reduction takes place on defect sites at the nanorod's surface, thus controlled etching allowed exposing more defect sites.^75^Fig. 3 presents this trend of increasing surface decoration with Au NPs. In the presence of air and trace amounts of water, etching of the NR provides additional nucleation sites for the metal, resulting in multiple non-epitaxial gold domains. Without exposure to air, only a single tip forms; with air, Au/CdS heterostructures are formed on both tips resulting in hybrid nano-dumbbells at reaction times < 90 min (panel e, Fig. 3); longer reaction times result in growth throughout the rod. An important consequence of even longer reaction times is a ripening process, where small Au domains dissolved back into the solution and were redeposited on the tip, leading to a single large Au tip.^75^

Using a similar procedure with a platinum source does not work. To achieve selective Pt-tipped CdS NRs, a combination of oleic acid (OA), oleylamine (OY) and 1,2-hexadecanediol in diphenylether is used. 10 min at 65 °C is sufficient to allow dissolution of the Pt acetylacetonate (Pt(acac)_2_) precursor, which is then injected at 200 °C into the CdS NR mixture.^76^

This procedure, adapted from a Ni_*x*_Pt_1−*x*_ synthesis,^77^ also allowed the formation of bimetallic tips (*i.e.*, PtCo and PtNi) when the other metal precursors are present in the system prior to injection.^76,78^ The choice of stabilizers is responsible for the metal (or bimetallic) tip—with the OA : OY combination being responsible for the spherical single-crystalline nature while the diol is mainly responsible for the reduction. Schlicke *et al.* have shown that a Pt-tipped CdS NR can be transformed in a subsequent step into a faceted metallic tip, by introduction of CO(g) to the growth solution in benzyl ether.^79^ The CO served a dual role—it reduced the Pt(acac)_2_ on the pre-formed Pt-tip and also dictated the final {100} faceted tip morphology.

**Fig. 4:**
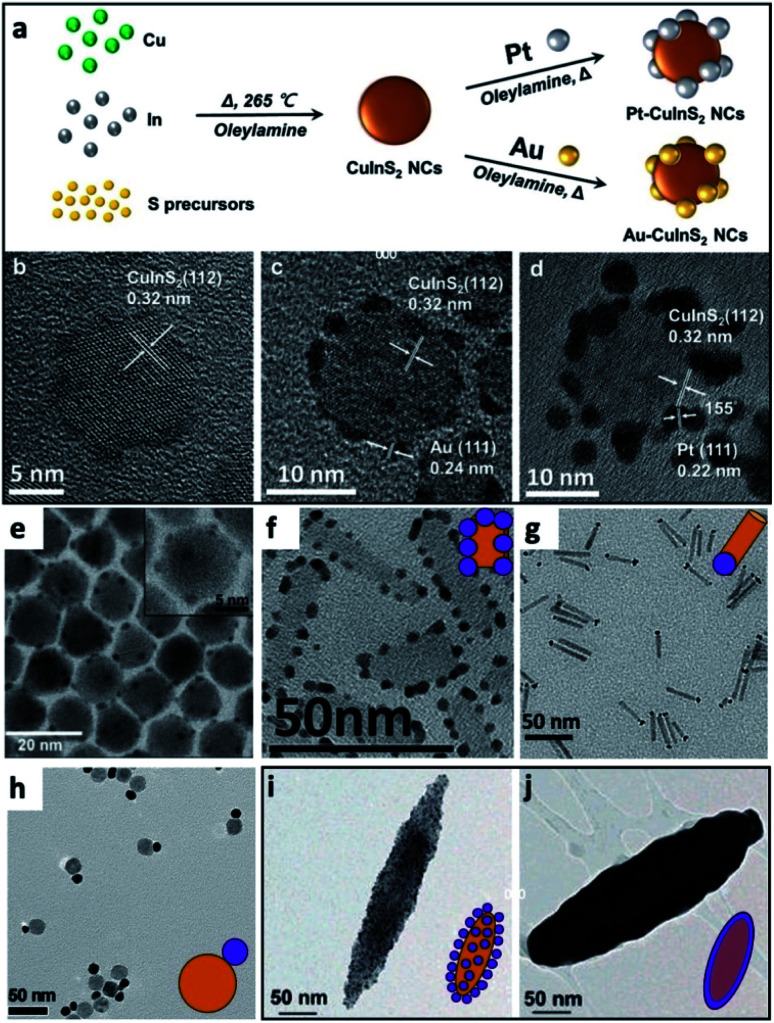
Gallery of chemical reduction and related methods of depositing metals on SCs. (a–d) Non-selective reduction of gold and platinum in the presence of OY on CuInS_2_ NCs, reproduced with permission from ref. 98, Wiley-VCH Verlag GmbH & Co., copyright 2019: (a) synthetic scheme, HRTEM image of (b) CuInS_2_ NCs, (c) Au/CuInS_2_ and (d) Pt/CuInS_2_. (e) CdSe NC/Au, where Au domains were reduced selectively on the apexes of the CdSe nanopyramids, reproduced with permission from the Royal Society of Chemistry, ref. 80 copyright 2010. (f) Pt on the edges of CdSe nanoplatelets (NPLs), adapted with permission from ref. 93, American Chemical Society, copyright 2015. (g) Selective reduction of Pt on one tip of CdS NRs, adapted with permission from the American Chemical Society, ref. 90 copyright 2016. (h) Au/Fe_3_O_4_ dimer, adapted with permission from ref. 108, American Chemical Society, copyright 2019. (i and j) Hybrid Fe_2_O_3_ ‘nanorice’ with Au NPs attached *via* a linker, (i) before and (j) after formation of a complete shell, adapted with permission from ref. 109, American Chemical Society, copyright 2006.

Some examples of different adaptations of the above-mentioned selective deposition methods of gold and platinum resulted in CdSe pyramids/Au,^80^ PbS QDs/Au,^81^ PbSe QDs/multiple-domain(s) and sizes of Au,^82^ PbTe nanocubes/Au,^83^ Ag_2_Se/Au and Ag_3_AuSe_2_/Au,^84^ Cu_2_ZnSnS_4_ (CZTS, a Cd-free SC with *E*_g_ ∼ 1.5 eV) cubes/Au and /Pt,^85^ NRs/Au,^86^ core@shell or heterodimers with Pt, Pd and Au,^87^ QDs/AuAg,^88^ CuInS_2_/Pt,^89^ CdSe@CdS NR/Pt,^90^ CdSe@CdS NR/Co,^57^ CdSe@CdS NR/Ni,^91^ Bi_2_S_3_/Au nano-dumbbells,^92^ anisotropic quasi-2D CdSe nanosheets or nanoplatelets (NPLs)^70,93–96^ and related 2D cadmium chalcogenides (*e.g.*, CdS^96,97^ and CdSe–CdS core–crown NPLs^94,95^) with metals such as /Au,^70,93–95,97^ /Pt,^93–97^ /Pd,^93^ metal alloys such as Pt–Au^95^ and Ni–Pt,^97^ as well as selective deposition of distinguishable Pt and Au domains.^95^ The mentioned variations include changes to the shape, size, morphology and material of the starting SC, as well as expansion of the deposited metals and alloys, some of which are reproduced in Fig. 4.

A case where no selectivity is required is the deposition of multiple metal domains (such as Au and Pt) on isotropic NCs (such as CuInS_2_); such an example from Tang *et al.* is shown in Fig. 4a–d, where only OY is used for reduction of both metals, and the authors integrated this HNS into a photodetection device after forming an additional interface with MoS_2_.^98^ Other examples are deposition of 10–20 nm tin domains on CdSe 2D NPLs in tetrahydrofuran with tetrabutylammonium borohydride as the reducing agent.^99^ The same procedure on CdTe NPLs does not result in distinct Sn domains, but rather multiple decorations accompanied by the formation of CdSn_3_Te_4_.

An alternative to the previously described large Au-domains was reported utilizing a combination of the ‘spontaneous’ Au nucleation (chemical reduction of AuCl_3_ in the presence of DDAB using octadecylamine) and a second photoreduction step. The first step results in uniform single-Au-tipped HNSs, which are subsequently cleaned and redispersed under an inert atmosphere. Then, a photoreduction of Au^3+^ cations on the Au tips (formed at the first stage) of the HNS occurs, with the Au domains serving as the nuclei, thus preventing the deposition of gold elsewhere.^100^ This two-step method allows for excellent control over the size of the tips. The previously mentioned report on Au functionalization of CdSe-based NPLs from Mahler *et al.* also elegantly shows the benefits of choosing the reduction mechanism to control the formed domains: chemical reduction of AuCl_3_ in the presence of DDA and DDAB at rt results in small Au tips (<5 nm) at the edges, preferably at the corners; switching to a photo-assisted reduction, where CdSe/Cd_0.5_Zn_0.5_S NPLs are reduced in the same environment but at 0 °C and under Xe lamp illumination, results in larger 10–20 nm Au domains; Furthermore, the same procedure in the dark, but *ca.* 70 °C (*i.e.*, thermal reduction) forms a large Au domain(s) (also) at the middle of the S-rich surface of the NPLs, with evidence of partial cation exchange (*i.e.*, an Au_2_S phase is detected alongside the Au domain).^70^

Other metals such as cobalt were deposited on CdSe NRs by heating a solution of an organometallic Co-precursor in toluene in the presence of lauric acid and hexadecylamine under a reducing H_2_ atmosphere.^101^ When a multielement SC is desired, an attractive synthetic path is the use of single-source molecular precursors (SSPs), which decompose to yield, for example, tetragonal or hexagonal CuGaS_2_ (ref. 102) and CuGa_*x*_In_1−*x*_S_*y*_Se_2−*y*_.^103^ At high temperature, when the SSP decomposes, hot injection of a gold source (HAuCl_4_) in OY yields the SC/Au hybrid.^102^

An alternative approach, which circumvents using organometallic precursors is the protocol by Yang *et al.*,^82^ where different aqueous metal salt solutions are mixed with an ethanolic solution of a long chain amine (*i.e.*, DDA), which allows transfer of the metal cation to an organic phase. They have used this to synthesize metal NPs, metal-sulfide SC nanocrystals, as well as metal-sulfide–metal HNSs. Such a multi-phase approach for metal NP synthesis has since been expanded for deposition of metals on non-sulfide-based SCs such as Pt–CeO_2_.^104^ An alternative from the Ryan group allowed controlled deposition of Au and Ag tips on Cd-chalcogenides (after sonicating their organic dispersions with octylamine and dimethylphenol, respectively) by mixing their precipitates (after centrifugation) in the respective aqueous metal-ion containing solutions, effectively inducing a phase-transfer.^105^ If the CdS NRs are water-soluble from the beginning, aqueous reduction of the metal is possible, as in CdS/Pt, but this approach is more suitable for coprecipitation of non-precious metal hydroxides such as CdS/Co(OH)_2_.^106^ A related mechanism involves the reduction of gold on the surface and/or edges of hydrophobic OY-capped CdSe/CdS NPLs by compression on a Langmuir–Blodget trough using an aqueous chloroauric acid subphase.^107^

There are also cases where a metal cation from the core can reduce a metal in its vicinity, for example, water-soluble Fe_3_O_4_ NPs in a boiling aqueous solution transform into Fe_3_O_4_/Au (shown in Fig. 4h) or /Ag, /Pd and /Pt heterodimers or metal-decorated Fe_3_O_4_ since Fe^2+^ on the surface can reduce Au(iii) to Au(0).^108^

Another strategy for forming SC/M HNSs is chemical anchoring. A covalently attached molecule to the SC, with an appropriate Lewis base moiety such as an amine or sulfide, can donate its electrons to colloidal metal NPs, thus initiating a self-assembly process. This approach was used by the Halas group, for example, to attach 1–2 nm Au NPs to SiO_2_ cores with a 60 nm radius, treated with an organosilane (3-aminopropyltriethoxysilane), which leaves the silica spheres with outbound amines that attach to the colloidal Au NPs. As a second step, to achieve complete metal coating, further reduction by NaBH_4_ in the presence of chloroauric acid and potassium carbonate was used to deposit gold on and between the anchored colloidal gold particles, which serve as the seeds. These steps allow control over the shell thickness in the range between 5 and 20 nm. A similar procedure also allowed formation of water-dispersible Fe_2_O_3_ dielectric core–Au shell rice-shaped NPs (Fig. 4i and j) for investigation of plasmonic phenomena when the thicknesses of the cores and the shell are controlled (the main difference being that the reduction step was catalyzed by the Au seeds and performed using formaldehyde).^109^ Chang *et al.* used surface modification of CeVO_4_ nanosheets to induce a self-assembly process with a thiol (–SH) functionalization (alongside transforming the NPs into hydrophilic ones). These sites allow Ag^+^ ions in the solution to be directly reduced by the Ce^3+^, forming a CeVO_4_/Ag interface.^110^

#### 2.1.3 Metal NPs as seeds for growth of semiconductors

**Fig. 5:**
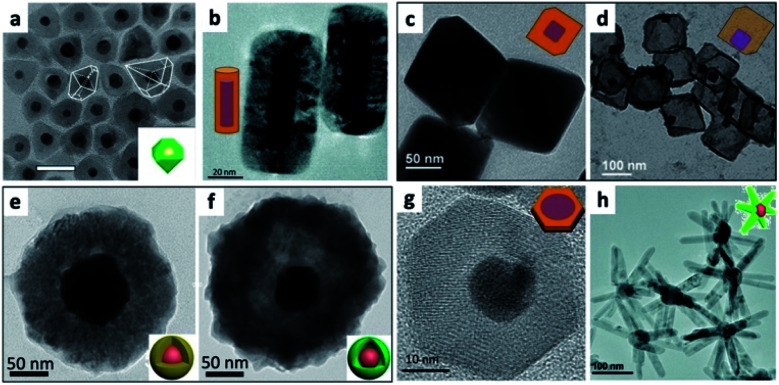
Examples of metals as seeds for SC overgrowth. (a) Au@NiS_*x*_ core@shell HNS, reproduced from ref. 127 with permission from Elsevier, copyright 2020. (b) Au NRs@Cu_2_O, adapted with permission from ref. 113, the Royal Society of Chemistry, copyright 2016. (c and d) Cuprous oxide overgrowth on Pd NP cores, where the amount of reducing agent controls the polyhedron morphology, in (c) Pd@Cu_2_O truncated octahedron, which after addition of Na_2_S and acidic treatment is converted into Cu_2_S and partly leaches out resulting in a Pd–Cu_2_S yolk–shell (d), adapted with permission from the Royal Society of Chemistry, ref. 117 copyright 2013. (e and f) Cu_2_O overgrowth on Au by reduction of Cu^2+^ with hydrazine in the presence of polyvinylpyrrolidone (PVP) (e); sulfidation converts the shell into Cu_9_S_8_ and hollowing begins (f), adapted from ref. 126 with permission from Wiley-VCH Verlag GmbH & Co., copyright 2019. (g) Au/CuInS_2_ disc, adapted from ref. 129 with permission from the American Chemical Society, copyright 2016. (h) ZnO multipods grow out of a gold core, adapted with permission from the Royal Society of Chemistry, ref. 132 copyright 2016.

Using metals as the first step for the overgrowth of semiconductors (described as part of approach (I) in Fig. 1) is a standard method that can result either in a variant of an M@SC core@shell system or partial coverage of the metal with semiconductor domains (*i.e.*, multiple heterostructures). Formation of core–shell systems is more challenging than heterostructures primarily due to the large lattice mismatch between metals and common semiconductors such as metal-oxides and other-chalcogenides.^111^ Huang and co-workers have pioneered the preparation of a variety of different Au core@Cu_2_O shell structures with the gold core dictating the achievable morphologies of the Cu_2_O shells. These included forming truncated triangular prisms with triangular Au plate cores, octahedral Au@cuboctahedral Cu_2_O, pentagonal prism coating of Au nanorods and others.^111^ In the same work and in following reports they further evolved the synthesis by introducing selective etching of Cu_2_O to form more complex exposed facets on these HNSs, such as star-shaped columns^111^ and face-raised octahedra with V-shaped {111} edges.^112^ Such M–Cu_2_O core–shell structures^113^ were extended to metals other than gold, *e.g.*, Pd,^114^ Au–Ag^115^ and Au–Cu^116^ cores by Huang, Pt, Pd and Ag by our group^117^ and epitaxial Au–Ag or Ag cores–Cu_2_O shell by Wang;^118^ see some examples in Fig. 5.

The interaction of the metal core and the controlled Cu_2_O shell thickness are responsible for modulation of the metal plasmon position, as well as exhibiting changes to the absorption position of the cuprous oxide (a p-type direct-bandgap oxide SC, *E*_g_ = 2.17 eV).^119^ Shevchenko *et al.* have shown modulation of the Au plasmon following coating with an iron oxide shell; a similar effect was shown using a cuprous oxide shell. When an anisotropic Au NR was used as the core, not only the longitudinal but also the transverse plasmon was shifted.^117^

Additional optical response tuning can be achieved through formation of hollow centers in a HNS, *e.g.*, Au@Cu_2_O core@shell,^120^ selective oxidation of Au NRs@Cu_2_O,^121^ formation of a selective gap between the outer metal core and internal Cu_2_O shell in Au NR@Ag@Cu_2_O HNSs^121^ and controlled formation of gaps in an Au–Cu_2_O yolk–shell system.^122^ For further information regarding the various LSPR (localized surface plasmon resonance) influences of the M–Cu_2_O and related M–metal oxide systems with emphasis on the facet-dependent properties we refer the reader to a review by Huang.^123^ On top of the tunable optical properties, these M–SC systems have potential catalytic applications, once the mechanism of electron transfer is understood. Wu and co-workers have explored the possible modes of electron transfer in Ag–Cu_2_O^124^ and in Au–Cu_2_O systems with a controlled silica insulating barrier layer, *i.e.*, Au@SiO_2_@Cu_2_O.^125^ The increased charge-carrier generation in Cu_2_O stems from direct electron transfer as well as plasmon-induced resonant energy transfer.

Controlled conversion of the oxide to a sulfide results in etching of the interior part of the shell, without hurting the metal core, for example, resulting in Au NR–Cu_2_S and Pd–Cu_2_S yolk–shell HNSs^117^ or Au@Cu_9_S_8_ (converted from Au@Cu_2_O core@shell by exposure to NaHS, tested also *in vivo* for photoacoustic imaging and photothermal therapy, Fig. 5e and f).^126^

Some additional examples where a metal seed is used include: Au/NiS_*x*_ where Au is the core at the center of a nickel sulfide polyhedron;^127^ Au/Fe_7_S_8_ NPL;^128^ Au/CuInS_2_ disc, where an epitaxial relationship was achieved by reacting Cu(acac)_2_ and In(acac)_3_ with DDT (thiolated Cu(ii) and In(iii) precursors) and OY in the presence of OY-capped gold seeds to first nucleate CuInS_2_ forming twin dots that evolved during the reaction at 200 °C to an epitaxial 0D fcc (cubic) gold/2D wurtzite (hexagonal) CuInS_2_ disc (Fig. 5g);^129^ Au/Fe_*x*_O_*y*_ formed by reacting Fe(CO)_5_ with OA, OY and Au seeds, followed by a subsequent carving of the gold domain using iodine;^130^ Au/ZnO;^131^ Cu/ZnO, where the zinc precursor, ligands and solvent allow formation of ZnO multipods (Fig. 5h), shell and pyramid over Cu NPs and nanoforest sheath over Cu NWs.^132^

Gordon and Schaak have shown how a starting Au NP can evolve into an Au–In_2_O_3_ heterodimer without nucleation, but rather by forming an Au–In alloy, which transforms into AuIn_2_ intermetallic NPs surrounded by amorphous indium oxide, evolving into the final product *via* phase-segregation.^133^ This case is an excellent example for comparing the mechanism to the general approaches described in Fig. 1: in contrast to approach (I), where we would have expected an indium-based SC to nucleate on the gold, here approach (III) is manifested. The intermediates could be analyzed by a careful planning of the reaction kinetics, achieved by temporal control over the indium precursor concentrations using a syringe pump. The indium alloys with the gold core until an intermediate AuIn_2_@amorphous InO_*x*_ forms (*i.e.*, approach (III) is at play, where InO_*x*_ precipitates out of the alloy core domain), first as a thin shell, and finally transforms into an Au–In_2_O_3_ heterodimer.^133^ The precipitation and heterodimer formation was induced by addition of an In(iii) precursor (In(iii) acetate in OA) into the hot alloy-core colloidal solution, thus possible oxygen sources are the acid moiety and the acetate of the indium precursor. The In oxidation can be regarded as a form of approach (II), which occurs during the synthesis.

#### 2.1.4 Chemical transformations resulting in M/SC hybrids

As was depicted in approach (II) in Fig. 1, HNSs can be formed by partial chemical transformation of the starting material. The two most common paths are oxidation and sulfidation of a metal (surface) to form metal/metal-oxide or metal/metal-sulfide interfaces, respectively, and partial (surface) reduction of a binary semiconductor to its metal, when the transformation is performed on a metallic ‘reactant’, *e.g.*, formation of Cu(0) particles over Cu_2_O films for CO_2_ reduction purposes,^134^ and cyclic voltammetry in the presence of Cl^−^ anions producing a chloride-stabilized biphasic Cu–Cu_2_O electrocatalyst from Cu_2_O.^135^

A partial oxidation can take place on various starting morphologies: for example, different spherical NPs,^136^ and nanowires (NWs, such as copper into cuprous oxide forming 1D Cu@Cu_2_O NWs).^137^ As discussed by Cozzoli and co-workers,^138^ centrosymmetric M–SC core–shell systems are most common with transition-metal cores (*e.g.*, Co, Fe, Cu) as they are easily oxidized. Several notable examples are mentioned herein: metallic copper NPs dispersed in hexane are oxidized under ambient conditions to form a Cu_2_O shell (the thickness of which depends on the elapsed time);^139^ this technique can be expanded to more complex starting NPs such as Cu@Ag—in which case, the benefit was the introduction of strain into the resulting Cu_2_O shell due to similarity with the Ag core's crystal structure;^140^ Pt–Co core–shell NPs can be oxidized to Pt–CoO by blowing an O_2_/Ar 1 : 4 v/v mixture into the solution at 455 K;^141^ Co is especially prone to oxidation as surface oxidation occurs even when the synthesis is carried out using standard air-free techniques.^142^ The study of metallic NP oxidation has consequences both on the synthetic opportunities of HNS design but also on the stability of NPs in applications and as an indirect characterization technique, as demonstrated by Ustarroz *et al.*, who studied the electrochemical oxidation of Ag NPs, which can result in dissolution (stripping).^143^

As was briefly mentioned in the previous section (Section 2.1.3, and will be discussed again in Section 2.3), sulfidation (also known as sulfurization) can transform a metal or a metal-oxide into a metal-sulfide, sometimes forming a void. It is, however, possible to partly convert a metal surface into its sulfide, thus forming a M–metal-sulfide HNS. For example, transforming a Cd metal core, on which ZnO NPs were grown, into a ZnO–CdS@Cd HNS;^144^ in this example—by treatment with Na_2_S(aq). Moreover, if a second metal shell is formed on the first M core before sulfidation, a complete conversion of the former leads to HNS formation, for example, Au/Ag NRs can transform into different Au NR core/(complete, corner-opened or end-opened) Ag_2_S shell HNSs.^145^

**Fig. 6:**
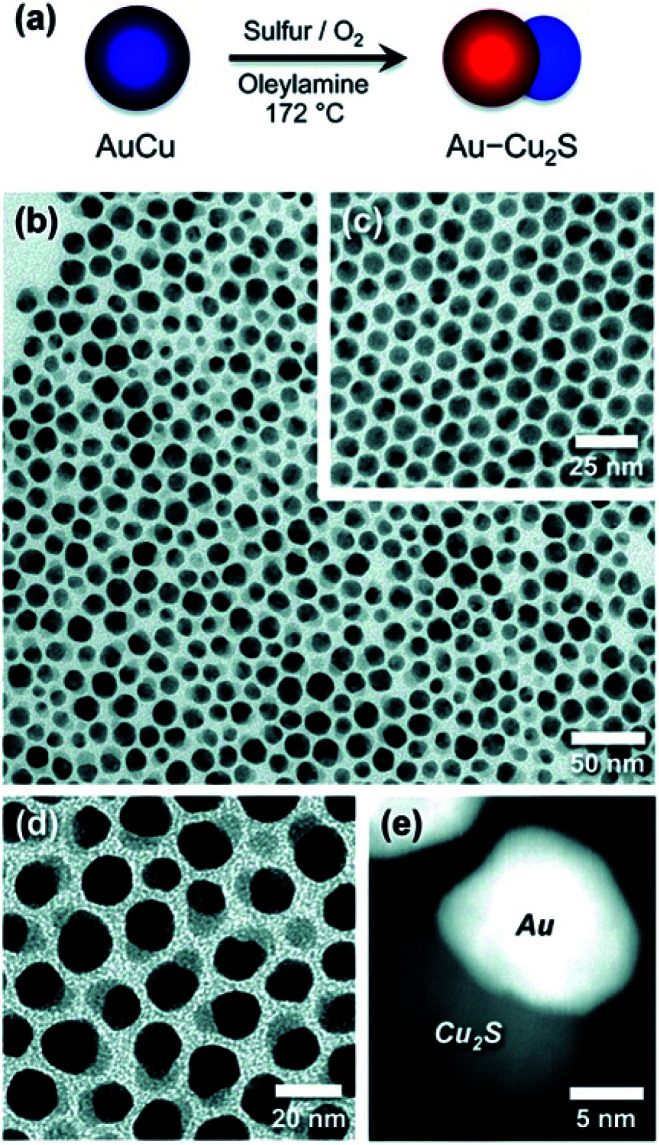
Chemically induced phase-segregation mechanism for the synthesis of Au–Cu_2_S heterodimers. In this mechanism the sulfidation does not partially convert the metal core—as depicted in approach (II) in Fig. 1—rather the bimetallic precursor phase-segregates into a dimer, with concurrent sulfidation of the copper into Cu_2_S, *i.e.*, combination of approach (III) in Fig. 1 and sulfidation. (a) Synthetic path scheme, TEM of (b) dimers, (c) AuCu NPs before synthesis, (d) higher-magnification of dimers, and (e) dark-field image. Reproduced with permission from ref. 146, American Chemical Society, copyright 2012.

Schaak and co-workers have designed bimetallic AuCu NPs, which were converted to Au–Cu_2_S when heated under bubbling oxygen in the presence of an OY surfactant and sulfur.^146^ This is an excellent demonstration of M–SC dimer formation without direct nucleation of an SC on the surface of the metal. In this case, depicted in Fig. 6, the dimer undergoes a chemically induced phase-segregation, where the copper transforms into a Cu_2_S SC. About 80% chemical yield is achieved, while only 3% of the particles seen in the TEM images are non-hybrid Cu_2_S NPs, which the authors suggest formed either through a de-wetting process of the HNS or by a reaction of the sulfur with a dissolved Cu-complex. The role of oxygen is to activate the transformation—probably by forming some kind of intermediate oxide. The authors have also checked whether an alternative mechanism of partial dissolution and reprecipitation is involved. Upon reacting Au NPs with copper and sulfur precursors, Au–Cu_2_S HNSs were formed but with yield *ca.* 16%—this different low yield suggests that the phase-segregation mechanism is the major reaction path.^146^

Silver is especially popular as the metal transformed into Ag_2_S.^147,148^ Han and co-workers, for example, formed various M–Ag metallic heterodimers (where M = Pd, Au, Pt). In a subsequent step, in the presence of PVP (polyvinylpyrrolidone) and Na_2_S the silver was sulfidized, resulting in a M–SC heterodimer.^147^ This effect can also happen unintentionally, as in the case of Ag NWs, which are sulfidized under environmental conditions.^149^

Furthermore, addition of another reactive precursor containing a different element can also form a ternary sulfide as demonstrated by van Embden *et al.*, who have used a ‘one-pot’ organic-phase synthesis (S coming from CS_2_, and DDT being the solvent) to form either core/shell or heterostructures of Ag/Ag_8_GeS_6_.^150^

A binary semiconductor can also be partially reduced to the metallic element it contains to form a SC/M interface. From the electronic structure point-of-view, even a reduction of few atoms due to charging (*e.g.*, Cd^0^ in a cadmium chalcogenide) has significant influence as it forms a trap.^151^ On a larger scale, an ammonia electrosynthesis catalyst was prepared by reduction of iron oxyhydroxide to form a core–shell αFe–Fe_3_O_4_ hybrid by annealing under a reducing H_2_(g) environment.^152^ Another interesting related example is the formation of a bimetallic thin layer in a HNS: Peng and co-workers have shown that for Pt/Fe_3_O_4_ core/shell triangular nanoprisms, an interfacial iron–platinum layer is formed.^153^ This serves as an epitaxial layer, and is therefore important as a possible synthetic tool to allow formation of heterojunctions using partial reduction of the SC.

Such an approach was also used to form hybrid bimetallic CdNi decoration of CdS NRs: in the first step CdS NRs were partially chemically reduced by dispersing calcined NR powder in an aqueous NaBH_4_ solution; subsequently, a photodeposition procedure on CdS/Cd NRs was performed in an ethanolic solution of nickel chloride salt, resulting in CdNi decoration of CdS NRs.^154^ Aqueous-phase reduction using NaBH_4_ was used to form a complex Ag/AgBr/BiVO_4_ photocatalyst.^155^ First, AgBr was precipitated over hydrothermally prepared BiVO_4_ microspheres from a solution of Ag^+^(aq) and Br^−^(aq). Then, silver NPs were deposited over the AgBr domains by reduction of silver cations. In such a mechanism the solubility product of the AgBr determines the available concentration of silver for the reduction step, effectively transforming an AgBr NP into an Ag/AgBr dimer.^155^

#### 2.1.5 Solution–liquid–solid (SLS)

The SLS growth mechanism (approach (III) in Fig. 1) was first reported by Buhro and co-workers for the growth of III–V semiconductor whiskers from indium.^156^ It has been expanded since for the formation of a wide variety of 1D (usually NW semiconductor) structures, *e.g.*, Ge, Si, InAs and other ME (M = In, Ga; E = N, P, As) as well as metal-sulfides MS (M = Pb, Cd) through the use of SSPs with controlled aspect ratios and monodisperse diameters.^157–163^ The SLS mechanism involves: (i) a reaction of metallo-organic precursors in a hot solution (the solution phase), usually initiated by a thermal decomposition; (ii) semiconductor components, which are formed from the precursor reaction, dissolve inside molten metal nanodroplets (the liquid phase) until supersaturation is achieved; (iii) the semiconductor crystal (the solid phase) starts to precipitate out of the metal. As the feed of new atomic components into the metal droplet is sustained, the supersaturation conditions are maintained, and the semiconductor continues to precipitate out. Since the activation barrier for semiconductor crystallization is lower at the liquid–solid interface, the precipitation of the semiconductor tends to preferentially proceed at this juncture, leading to 1D growth behavior. As a result, the SLS process usually results in 1D structures such as nanowires and nanorods. The metal nanodroplets play two mechanistic roles—they catalyze both the decomposition of the precursors at the solution–liquid interface and the semiconductor growth (precipitating out of the droplet) by functioning as a crystallization solvent.^164^ A strong correlation was found between the diameter of the metal droplet and the diameter of the growing NW (*e.g.*, Au droplet and PbS NW).^161^ Therefore, the mean size of the metal nano-catalysts and their size monodispersity are crucial. SLS-grown CdSe NWs (from a Bi catalyst) can act as the nucleation sites for further metal deposition (overgrowth) of metal or binary-metal domains (Au, Pt, PtCo and PtNi).^165^ In this mechanism the main means of control over the deposition is the interplay between the concentrations of the NWs and the metallic precursor.

**Fig. 7:**
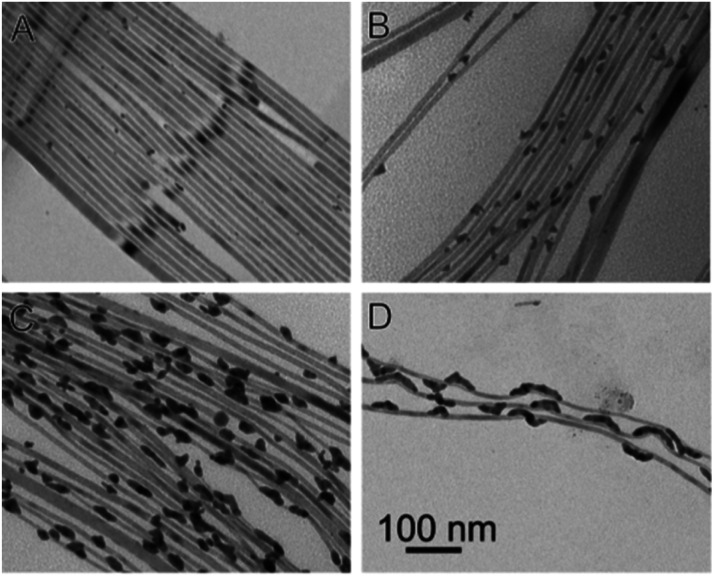
Gold overgrowth over SLS-grown CdSe NWs.^165^ Increasing amount of Au precursor from (A) to (D). Reproduced with permission from the American Chemical Society, copyright 2009.

Reduction of Au(iii) using DDA (a low AuCl_3_ concentration as the reactant source) in the presence of CdSe NWs and DDAB surfactant results in predominant growth at the tips (Fig. 7A). Increasing the Au/NW ratio results in enhanced overgrowth, probably on surface-defect sites. Further increase of concentration changes the mode of growth, with a transition from individual growth sites to areas of continuous overgrowth (Fig. 7C). At the highest concentrations, the integrity of the CdSe NW itself is endangered due to severe strain originating from the lattice mismatch of wurtzite (hexagonal) CdSe and cubic Au.^165^

From the synthetic point of view, an important feature to achieve monodisperse size distribution of the CdSe NWs is the need for homogeneity of the Bi catalyst for the SLS mechanism. In the latter example, it was achieved by using a thin film of Bi deposited on a Si substrate, which melts and forms uniform droplets as had been reported by Wang and Xia.^166^

#### 2.1.6 Heterostructuring constraints: kinetic barriers and thermodynamic aspects (*e.g.*, ligand type and coverage, crystal structure, geometry and surface energy)

A good example for the effect of surface energy due to geometric considerations is a heterostructure where a nanopyramid is involved. Au–ZnO nanopyramids were formed by several methods including photoreduction. Yao *et al.* used a ZnO nanopyramid solution (a preliminary solvothermal synthesis) in the presence of HAuCl_4_ under UV irradiation to form single-tipped Au–ZnO nanopyramids.^60^ This hybrid has a polycrystalline Au NP tip, which is selectively formed at the pyramid's vertex, and always as a single NP per ZnO pyramid. The pyramid's growth direction is the *c*-axis of ZnO, *i.e.*, the [001] direction is perpendicular to the pyramid's base. Calculations show that the LUMO includes localized Zn sp orbital character, which is located at the pyramid's vertex. Thus, upon photoexcitation, electrons transfer to the (001)-Zn surface at the vertex, where they readily reduce the Au cations.

A recent study from the Waclawik group demonstrated a colloidal synthesis of different ZnO NC morphologies with a labile and relatively weakly bound ligand—benzyl amine. They have studied gold photodeposition on four different morphologies, in EtOH or EtOH : toluene mixture as the solvent (sometimes deaerated), with pulsed or continued irradiation.^167^ Photolysis of AuCl_4_^−^ using UV illumination produces a metastable Au(i) complex [AuCl_2_]^−^, which in turn is reduced by excited electrons from the ZnO NC.^63^ They have shown that adjusting the described parameters allowed control over the average Au NP(s) per ZnO, as well as facet selectivity. They conclude that the most important factors are the charge distribution along the surface of the excited ZnO NC (influencing factors: shape, solvent and dissolved oxygen concentration, irradiation technique) and the energy barrier to nucleation at potential nucleation sites, which are determined mainly by the crystal facet and defects therein.^167^

Chen *et al.* have shown that by synthesis of ZnO nanoflowers using zinc stearate in a suspension of Ni@Au with OY, dibenzylether and hexadecanol they could form a hybrid that exhibits preferential orientation between the metal core (*ca.* 14 nm) and the ZnO pyramid (each ∼28 nm petal of the nanopyramid is a triangular pyramid).^168^ The Ni (111) plane is parallel to the wurtzite (0001) plane, which is a form of homoepitaxy, with most flat Ni facets acting as the nucleation and growth sites for the ZnO.

An important example for the thermodynamics of such systems was shown by the authors' group, where metal (Cu, Ag or Au)–ZnO nanopyramids with a selective metal attachment to the base or the tip of the pyramid were synthesized. In both cases, a one-pot reaction was carried out in the presence of oleic acid and oleylamine, which are the reducing and capping agents.^169^ When ZnO pyramids were present in the solution during the reduction of metal monomers, selective growth on the tip occurred (‘tip-attached’); when the precursors for both materials were heated-up, ‘base-attachment’ occurred since ZnO grew over the faster-forming metal NCs. This demonstrates the importance of the reactive sites. Once nanopyramids were present in the growth solution, the metal tended to nucleate on the vertex, due to the high surface energy of this geometry.^58,72^

As was described for these M/ZnO systems, there is a large mismatch between the lattice distances, which prevents formation of complete anisotropic core–shell systems. One way to circumvent this limitation is using a hydrothermally prepared coating of TiO_2_ over Ag NW cores, which allows deposition of multiple ZnO NPs as the sheath.^170^ Since Ag NWs have potential application as the main ingredient of conductive transparent (flexible) electrodes, such hybrids are of much interest, and simple deposition techniques (spin coating and mild thermal annealing) were used to this end to form ZnO NP/Ag NW composite mesh over polyethylene terephthalate (PET) as an example.^171^ Since the outcome is attachment based on random physical interactions without controlled interfaces, we will not discuss this synthetic approach further.

The previously discussed, Au-tipped Cd-chalcogenide system provides further insight into the formation of asymmetric hybrid structures from symmetric ones.^72–74^ Despite the symmetry of CdSe NRs in a colloidal solution, the gold complex is attracted to the tips. This stems from the combination of the following: (i) the tips are a location with a strong curvature, thus they have a high specific surface free energy (*γ*), relative to the sides of the NRs, (ii) the surfactant passivation at the tip is less ‘dense’, with higher defect probability both due to geometric considerations and the different exposed crystal plains (which during the high-temperature growth of the wurtzite hexagonal CdSe phase of the NRs grow parallel to the *c*-axis, *i.e.*, 〈001〉). For these reasons, there is both a kinetic tendency for reduction on a tip (high number of Au-complexes adsorbed at the tip, and others with relatively easy access) and a thermodynamic one (*γ*). It is worth mentioning that removing part of the passivating NR ligands prevents the selective growth at the tips as in the report on the growth mechanism of Co on CdSe NRs by the Alivisatos group.^79^ Another consideration for single metal tip formation was demonstrated in a CoPt/CdS NR system. Here, unlike Pt under the same reaction conditions, Co does not nucleate on the CdS NR without an existing Pt tip, as its precursor is not reactive enough.^78^ In this mechanism, a Pt tip is formed (which could also be aided by the Pt–S interaction to the CdS at the tip) before a subsequent Co co-alloying occurs. In contrast to bimetallic alloy NP formation in solution, where each NP has a uniform Co and Pt distribution, in this case, a complete alloying of the Pt tip with Co takes place (fcc solid solution), but as the tip grows, strain release becomes possible by Co segregation or enrichment of Co at the later stages of tip growth.

The other point that needs explanation is why a low concentration of gold usually results in deposition on a single tip, forming an asymmetric structure. This is explained by the crystal structure of the NR that has one Cd-terminated end while the other end is chalcogenide-terminated. The electron-rich chalcogenide facet is expected to have higher reactivity towards gold reduction.^74^ The formation of asymmetric structures is not limited to the initial stages of metal deposition. With increasing concentration, after both ends of the NR are with Au tips (nano-dumbbell), a ripening process occurs, where small Au domains dissolve (due to both higher specific surface free energy and easier oxidation of small Au domains).^172^ This is an electrochemical ripening process that benefits further growth of the larger Au domain, since the free electron from the dissolving small domains migrates through the NR to the electron sink.

To achieve a metallic gold shell over CdSe dihexagonal pyramidal nanostructures, Meyns *et al.* have used wurtzite CdSe NCs as the seeds for a modified AuCl_3_ reduction procedure.^80^ They have reacted the NCs in the presence of OY and an Au(iii) complex resembling Au(iii)–DDAB (specifically, AuCl_3_ with *n*-dodecyltrimethylammonium bromide, DTAB). Under these reaction conditions, the OY surfactant is a mild reducing agent and a thin (partial) amorphous gold shell was formed around the CdSe NCs. When irradiated under a TEM electron beam, this shell transformed into Au dots on the vertices of the NC. This observation demonstrates several important lessons: (i) it shows the importance of high *γ* locations on a NC—when the reaction conditions allow a metastable state to reach the most thermodynamically stable one—high-energy vertices are classical nucleation points; (ii) crystal structure, morphology and ligand-passivation play a crucial role—when CdSe NRs of the same crystal structure are subjected to the same procedure, the result is the established double Au-tipped CdSe NRs. Since both morphologies have the same crystal structure, the empirical difference is attributed to (a) denser ligand coverage of the NRs, which poses a kinetic barrier to reduction on the NR's surface, thus the gold reduction occurs preferably on the tips (as discussed earlier); (b) despite sharing the same crystal structure with the NRs, the bipyramids have many Se- or Cd-terminated {101} surfaces, on which gold interacts with the Se (while the Lewis base moiety of the surfactant attaches to Cd atoms, which are Lewis acids). Additionally, a note of good practice is to remember that high-energy electron microscopy is not a ‘sterile’ environment and can induce not only beam damage but also chemical transformations, *i.e.*, reduction (and in fact, using a stronger reducing agent, gold dots were formed on the vertices during the synthetic stage).^80^

In a subsequent report, Klinke, Juárez and co-workers tackled the oxidation state of gold not as a post-modification but during the synthesis itself.^173^ They have elegantly shown that using Au(i) or Au(iii) complexes changes the final deposited gold morphology. They have switched the surfactant from alkylamine to dodecanethiol to easily obtain a solution of Au(i) precursor. Using the Au(iii) complex only (with a subsequent addition of surfactant for stabilization), a shell was formed. When on the other hand, they injected an AuCl_3_–DTAB complex mixed with the DDT, *i.e.*, an Au(i)-complex, the ‘standard’ gold dot nucleation occurred on several vertices of the CdSe NC.^173^ This report also explains the unstable gold shell as being composed of AuSe or AuSeCl (the Se source is the etching of the CdSe NC by the halide anions present).

For further discussion of the factors governing the formation of heterostructures *vs.* multi-component mixing, *i.e.*, formation of solid solutions during synthesis, we refer the reader to a review by Jeong and co-workers.^174^

#### 2.1.7 Complementary techniques that allow HNS formation and transformation

An exciting new pathway for control over the metal growth on a SC is the use of various soft templates. Weichelt *et al.* have shown that anchoring DNA functionalization of the surface of CdS NRs allows their placement in DNA origami, which in turn allows pre-determined and controlled deposition of metal.^175^ Lin *et al.* have recently shown the formation of hollow hybrids of Au or Pt with TiO_2_, ZrO_2_ and Ce_*x*_Ti_1−*x*_O_2_ and applied the Pt/ZrO_2_ hybrid as a stable catalyst for methane combustion.^176^ They have used a sol–gel based synthesis of metal oxides, forming a low-crystallinity oxide. They complete the first step by using urea and formamide polymerization. This step is a polymerization-induced colloid aggregation, which captures both the metal oxide and the metal salt. As a second step they heat the system, which crystallizes and hollows out the metal oxide spheres with metallic nanoparticles trapped within the (now crystalline) metal oxide shell.^176^

We have not explicitly discussed anionic and cationic exchange reactions^177^ of a semiconductor and galvanic exchange reactions of metals, but these are indirect synthetic paths to transform one domain of an HNS into another when the starting materials are more compatible and have a known proven syntheses. These reactions expand the possible attainable HNSs. For example, starting with a Pt–MnO heterodimer allows its transformation into Pt–MnE (E = S, Se), and finally to Pt–ME (M = Cu, Ag),^178^ or Zeng and co-worker's letter on Fe_3_O_4_/M mentioned in Section 2.1.2,^108^ where the aqueous-phase synthesis allowed further redox-based transformation of the metal (*e.g.*, Ag into an AgPt alloy, Au into an Au@Pd core@shell).

### 2.2 Transition-metal–nonmetal compounds and other materials

**Fig. 8:**
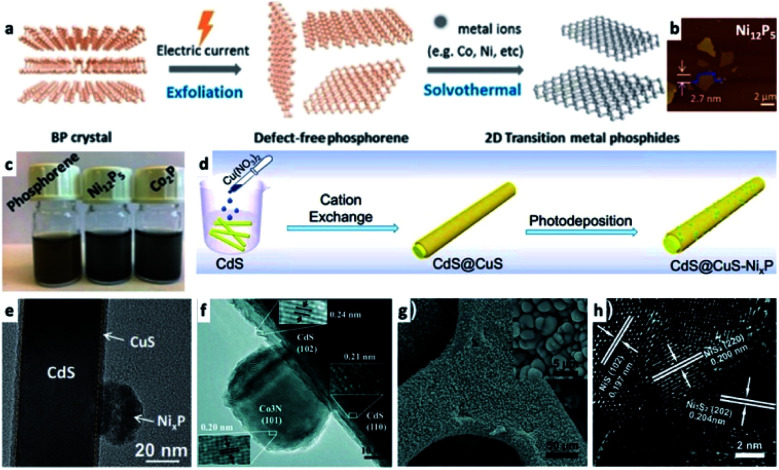
Examples of transition-metal (TM)–nonmetal compounds and their hybrids or chemical transformations. (a–c) Transformation of a 2D phosphorene into transition metal phosphides (TMPs) such as Ni_12_P_5_ and Co_2_P;^184^ (a) synthetic scheme showing that after an exfoliation step, a solvothermal reaction in the presence of TM ions forms the final product, (b) an atomic force microscope (AFM) characterization of Ni_12_P_5_, (c) images of vials with the dispersions. (d and e) A Ni_*x*_P TMP/CdS@CuS HNS; (d) a synthetic scheme where CdS NRs are coated with a CuS shell *via* cation exchange, followed by photodeposition of Ni_*x*_P, (e) TEM of this HNS, adapted from ref. 220 with permission from Elsevier, copyright 2019. (f) Co_3_N/CdS HNS formed by a hydrothermal reaction of cobalt nitrate and an amine source (hexamethylenetetramine) in the presence of CdS NRs, reproduced from ref. 207 with permission from the Royal Society of Chemistry, copyright 2017. (g and h) Ni_*x*_S/Ni HNS formed by sulfidation of a nickel foam substrate: (g) SEM, (h) HRTEM, reproduced from ref. 230 with permission from the Royal Society of Chemistry, copyright 2018. (a–c) Adapted from ref. 184 under a Creative Commons Attribution License, published by Wiley-VCH Verlag GmbH & Co., copyright 2020.

In the previous section a description of interfacing different 2D semiconductor materials with a metal junction (for example, Ni/CdS nanosheets and Pt/CdSe NPL, shown in Fig. 2c and 4f, respectively, or Ni NP decorating MoS_2_ (ref. 179)) was given. Such materials have relevance to transistor (opto)electronics, hence various interfaces are formed *via* non-colloidal methods.^180^ The strong van der Waals (vdW) interactions between 2D layered materials such as graphene,^181,182^ phosphorene,^183,184^ transition metal dichalcogenides (TMDC),^185,186^ graphitic carbon nitride,^187,188^ and hexagonal boron nitride (h-BN)^189^ allow numerous opportunities to interface 2D–2D materials. The vdW interactions are a fascinating synthetic approach as it can direct the integration of mixed dimensionalities ranging from 0D to 3D complex structures into a variety of devices and architectures.^180,190^ In the colloidal synthetic realm, usually an exfoliation step is required before interfacing with metals,^191^ metal cations (which may transform the 2D substrate, see Fig. 8a–c)^184^ or other material classes. We refer the reader to a review by Dubertret and co-workers on 2D colloidal nanocrystals.^192^

Other classes of interfaces receiving tremendous attention in recent years mainly due to their promising catalytic abilities are metal-containing molecular species,^193–196^ hydrogenase enzymes,^197^ metal–organic frameworks (MOFs),^198^ and even metal-encapsulated MOFs.^199–203^ Since their attachment to the SC is usually chemisorption or some physical interaction (electrostatic), they are out of the scope of this review. Recently, Wolff *et al.* have demonstrated that molecular Ru-containing cocatalysts can augment classical CdS nanorod/Pt hybrid systems and achieve water splitting without sacrificial substances.^204^

An account from the Eisenberg group relates the development of Co and Ni containing molecules as alternatives for Pt or Pd as the catalysts for proton reduction to hydrogen.^205^ Simply put, after achieving the described significant progress in homogeneous catalysis using Co- and Ni-complexes, inspired by the dye-sensitization of semiconductors such as CdSe QDs, they have investigated the catalytic enhancement of aqueous dispersions of CdSe QDs using Ni^2+^ and Co^2+^. This account demonstrates how knowledge transfer between homogeneous molecular catalysis and heterogenous nanoscale catalysis is fruitful. Ni and Co as representative examples are now used not only as constituent atoms in molecular surface modifiers, but rather are part of a wide range of transition-metal (TM) based binary and ternary materials.

The transition metal chalcogenides (mostly oxides and sulfides) and pnictides (nitrides and phosphides) are common cocatalysts for photo- and electro-catalysis. Some of them have a metallic character, for example transition-metal phosphides (TMPs), which can be viewed as P-doped metals (Ni, Co, Fe, Mo, Mn, Cu),^206^ and TM-nitrides (*e.g.*, Co_3_N, a metallic interstitial compound used in a Co_3_N/CdS NR HNS shown in Fig. 8f),^207^ while others can be semiconductors of varying bandgaps (*e.g.*, Co_3_O_4_ (ref. 208) and late transition metal monoxides such as CoO,^209^ which have size-dependent band positions^210^). Generally, the catalytic activity of cobalt oxides stems from charge transport properties and the valency of the metals in the crystals, which is analogous to molecular systems. An important example is the use of CoO as a hole acceptor from a photoexcited TiO_2_ nanostructure, where a spatially separated Pt domain simultaneously acts as an electron acceptor.^211^ Other examples include a heterogeneous Ni–O bond containing cocatalysts on CdS (/Ni(OH)_2_,^212^ /NiO,^213^ /Ni_2_O_3_ (ref. 66)) and related systems,^214–216^ which resemble homogeneous molecular Ni-catalysts adsorbed on CdSe for hydrogen evolution.^212^

TMPs^217^ are common as the metallic cocatalysts in colloidal SC/M systems. Hybrid systems with a light absorbing semiconductor such as CdS include CdS QDs–TMP (CoP, Ni_2_P and Cu_2_P)^218^ and CdS NRs–TMP (MoP,^219^ Ni_*x*_P and Co_*x*_P^220^), which were used for water reduction, while electrostatically assembled Co_3_O_4_ on CdS NRs were used as water oxidation catalysts.^221^ It is important to stress that not all phosphides have metal-like properties, and various semiconductors are constantly synthesized and their properties are investigated, for example 1D SnIP.^222^

In the field of electrocatalysis various sulfides, phosphides, nitrides, oxides and (oxy)hydroxides are common catalysts, which are deposited on a substrate that serves as the electrode and they enhance either or both the activity and stability (for example, due to higher stability than the Si substrate in a strong alkaline environment).^223–227^ A common starting high-specific-surface-area metallic substrate is nickel foam (NF), on which gas-phase electrodeposition and a variety of solvothermal reactions are used to deposit the metallic catalyst. Various redox catalysts were deposited on NF such as binary Ni-based materials, *e.g.*, Ni_2_P,^228^ Mo-doped Ni_2_P,^229^ ternary amorphous tungsten-doped Ni_*x*_P,^228^ or multiphase nickel sulfides,^230^ taking advantage of the abundant Ni surface. Electrodeposition allows forming CoPi over NiFe foam as well as Ni and Co oxides over NF.^231^

3D crystalline Co/amorphous Co_3_O_4_ core/shell nanosheets were demonstrated by a solution-phase deposition of cobalt oxide on the NF substrate, followed by partial reduction using hydrogen at 200 °C (where the newly formed Co crystals are the core of the amorphous Co_3_O_4_).^232^ More complex systems include NF supported 1D/1D hybrids of Ni_12_P_5_ nanowires over Ni_3_S_2_ nanorods^233^ and Pt over NF that was transformed into a mixture of nickel phosphides *via* solvothermal phosphorization.^234^

Phosphorization is also common in the gas-phase, for example by decomposition of NaH_2_PO_2_ at elevated temperature under a protective Ar atmosphere to transform a Ni/Cu/polymer into Ni_2_P–Cu_2_P@NiCuC,^235^ or trialkylphosphines over different metal foils, under 5% H_2_/Ar flow to eliminate surface oxides.^236^ In another recent phosphorization example, In-doped cobalt oxide was transformed into CoO/CoP for OER application in metal–air batteries.^237^ Another approach is formation of a hybrid structure which uses low amounts of precious metal (for example, Co_2_P/Pt core/shell NRs),^238^ and subsequently deposit it on an electrode, possibly in the form of a carbon matrix.

Recently, novel approaches have been utilized for the formation of 3D porous substrates *via* metallization, for the subsequent transformation into metal phosphides *etc.*, for example using virus templates to form Ni_3_P or Ni_2_P and Ni_5_P_4_.^239^

This class of materials are treated in this review as semiconductors due to the similarity in the synthetic methods to other binary and ternary semiconductors as demonstrated in the work of Shavel and co-workers who have synthesized various colloidal TMPs of different aspect ratios.^240^

### 2.3 The HNS as a platform for hollow NPs

**Fig. 9:**
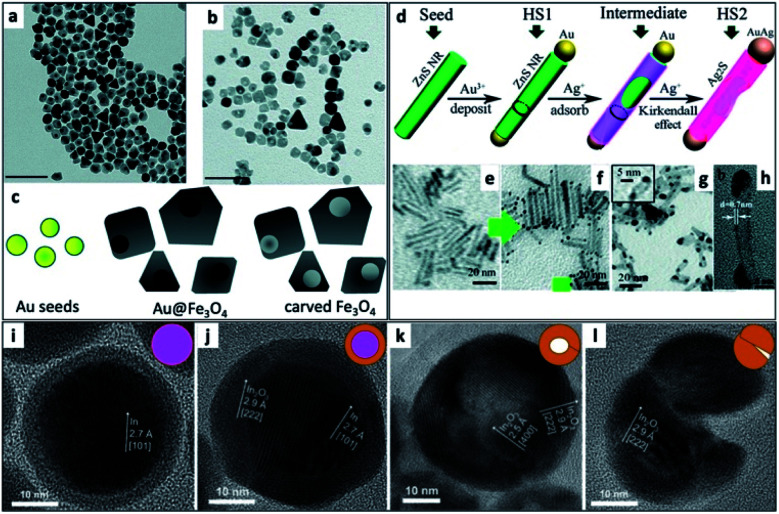
Hollow nanostructures derived from HNSs. (a–c) Au NPs serve as seeds for the growth of a shell (Au@Fe_3_O_4_, TEM shown in (a)), subsequently etched with iodine, removing the gold and resulting in carved Fe_3_O_4_ (b); scale bars are 100 nm; (c) scheme.^130^ (d–h) The nanoscale Kirkendall effect is responsible for the formation of Ag_2_S nanotubes with a gold–silver tip: (d) the synthetic scheme, (e) ZnS NRs (*d* ∼ 3 nm) serve as seeds for the reduction of gold on its tips (HS1, shown in (f)); following the exposure of HS1 to Ag^+^ ions two phenomena occur—the Au tip is alloyed to form an AuAg tip and the rod is transformed *via* the Kirkendall effect (silver cations diffuse inside, replacing the zinc cations, which diffuse outside) resulting in a hollow silver sulfide nanotube, Ag_2_S NT/AuAg (HS2); confirmation of the mechanism is evident from TEM images (g), where *d* ∼ 5 nm; a wall thickness of 0.8 ± 0.1 nm is confirmed by (h) HRTEM.^243^ (i–l) Hollow indium oxide NPs are formed *via* a non-Kirkendall mechanism in a deaerated atmosphere,^249^ namely In NPs (i) with a native amorphous InO_*x*_ shell are first thermally oxidized to form an In@In_2_O_3_ core@shell (j), additional heating is responsible for crack formation due to thermal expansion and void-formation, resulting in hollow In_2_O_3_ (k); some NPs exhibit significant cracking shown in (l); the inset of every TEM image contains a schematic cartoon of the NS. (a–c) Adapted from ref. 130 with permission from the Royal Society of Chemistry, copyright 2018. (d–h) Adapted from ref. 243 with permission from the Royal Society of Chemistry, copyright 2015. (i–l) Adapted with permission from Wiley-VCH Verlag GmbH & Co., copyright 2013.

An interesting synthetic path to achieve hollow nanostructures has been demonstrated by several groups involving types of core@shell morphologies.^241^ Hollow NP formation is especially easy to detect when the formation is performed *in situ* using the electron beam of an electron microscope.^242^ Alongside the previously mentioned selective metal etching (using I_2_) of an HNS, leaving voids in the SC (Fig. 9a–c),^130^ one of the most common methods to achieve hollow NSs is the nanoscale Kirkendall effect,^141^ where a void is formed in NPs due to a significant difference in diffusion rates. In their report, Yin *et al.* have first demonstrated a designed experiment that harnesses this effect on the nanoscale with Co NPs. They showed how cobalt is sulfidized upon addition of a sulfur solution into a pre-heated Co NP solution. The hollow Co_9_S_8_ (at different Co/S ratios, also Co_3_S_4_ or their mixtures) is formed because the Co core that leaches out through the CoS shell much faster than sulfur is able to diffuse from the exterior, through the formed shell, resulting in hollow CoS as a representative cobalt chalcogenide. Another example is the formation of Ag_2_S nanotubes from ZnS NRs while the metallic Au tip is transformed into a bimetallic AuAg tip (Fig. 9d–h).^243^

A ‘reversed’ nanoscale Kirkendall effect has also been reported, where Au-decorated an InAs HNS was used as the starting material for the formation of a crystalline metallic Au core/amorphous (oxidized) InAs shell with voids due to faster inward diffusion of Au in an InAs matrix compared to the self-diffusion of InAs (outward).^244^ An additional limiting case of a Kirkendall effect was exploited by Manna and co-workers, who have used the different susceptibility towards oxidation of the ingredients of a copper selenide core/copper sulfide shell nanocrystals.^245^ They showed how Cu^+^ ions diffuse into the solution upon exposure to an oxidizing etchant (CuCl_2_). Subsequently, Cu^+^ cations diffused outwards from the Cu_2−*x*_Se core through the Cu_2−*x*_S shell, thus forming various hollow particles, including collapsed NCs. Though not a regular M/SC HNS example, it shows how similar systems can be used to expand the scope of synthesized multicomponent nanostructures (*e.g.*, *via* cation exchange,^246,247^ and confined nano-to-micro particles).^241,248^

A related anion exchange phenomenon was exploited to form (hollow or void-containing) ZnS@Sn:ZnO nanostructures.^250^ Furthermore, different diffusion rates can be used to transform hybrid CdSe/Cu_3_P nanoplatelet systems into Cu_2_Se.^251^ Combination of such exchange strategies alongside selective etching was reported by Fenton *et al.* who formed various multi-domain nanostructures with combination of metal-sulfides, metals and voids.^252^

Another non-Kirkendall effect has been demonstrated using In@In_2_O_3_ core@shell NPs, which fractured upon heating to 250 °C due to the melting of the low-melting-point metal core. As a consequence, hollow indium oxide NPs were obtained (Fig. 9i–l).^249^

## 3 Formation of complex nanoparticle systems

### 3.1 Rational design of nanoparticles with increasing complexity

**Fig. 10:**
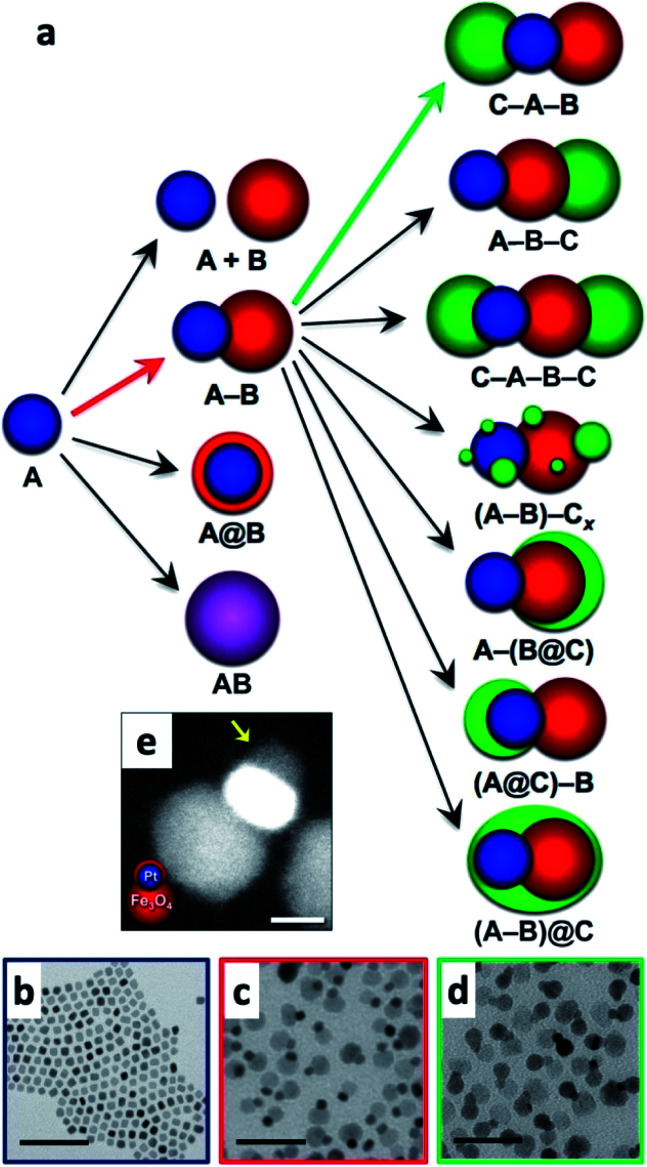
Sequential seeded-growth allows formation of various higher-order hybrid nanostructures, namely heterodimers and heterotrimers. (a) The first reacting NP is A (blue), which reacts with a second B NP (red), which in turn results in either a mixture of A + B, a heterodimer (A–B), a core@shell morphology (A@B) or an alloy particle (AB in purple implying a solid solution has been formed). The A–B heterodimer serves as the seed for deposition of a subsequent third material C (green).^253^ (b–d) TEM examples of the process depicted in (a) using color arrows: (b) Pt cubes serve as the seeds for formation of (c) Pt–Fe_3_O_4_ heterodimers (through the red arrow) that in the final step (through the green arrow) serve as seeds for selective gold deposition on the metal domain, forming the (d) Au–Pt–Fe_3_O_4_ heterotrimer.^254^ (e) Variation of A–B formation, where Fe_3_O_4_ forms a thin shell over the Pt in this heterodimer example with a protective layer.^256^ Adapted with permission from the American Chemical Society, (a–d) copyright 2017, (e) copyright 2014.

Multicomponent nanoparticles can be synthesized with multiple materials and domains, thus benefiting from two or more interfaces, at least one containing an M/SC junction. The final morphology can be a variant of a core@(multi-)shell, different multi-domain nanostructures and combinations thereof. Fig. 10a is a schematic representation of various multiple-heterojunction morphologies, which are attained from a single nanoparticle, starting with an ‘A’ NP, which can react with ‘B’ and consequently with ‘C’.^253^ In Fig. 10, some possible heterotrimer morphologies are depicted resulting from deposition of material ‘C’ on ‘A–B’ dimers that were formed in a previous step, adapted from Hodges *et al.*^253^ The Schaak group has thoroughly investigated the depicted heterostructures,^253–256^ and has even expanded the breadth of these methods, for example, by introduction of a solid-state protective group. Without a protective group (from A–B to C–A–B, marked with a green arrow in Fig. 10a), the third material (the metal) is formed on the metal of the dimer (TEM images in Fig. 10c and d show deposition of Au on Pt–Fe_3_O_4_ dimers, forming an Au–Pt–Fe_3_O_4_ heterotrimer).^254^ If, on the other hand, the dimer involves a thin shell, as is the case in the Pt@Fe_3_O_4_ example shown in Fig. 10e, where Pt is completely coated with Fe_3_O_4_, the subsequent Ag deposition occurs on the iron oxide, opposite to the Pt domain (without the protective group an Ag–Pt–Fe_3_O_4_ formed).^256^

An example of a core–multi-shell HNS is Au NRs, which are transformed into Au/AuAg to utilize the option of sulfidation, allowing the synthesis of Au/AuAg/Ag_2_S by reacting with thioacetamide in a basic environment. Finally, an additional shell layer of PbS could also be synthesized.^257^ A synthesis in the organic phase can also be used to form multi-shells as demonstrated by our group for M@ZnS@ZnSe (M = Ag, Au).^258^

Amongst the multi-domain HNSs, a system that was discussed previously and was explored in our group is the single-tipped semiconductor nanorods, where the metallic tip acts as an electron sink, thus serving as an excellent reduction catalyst (further discussion in Section 4.2). As SC NRs with a single metal tip have beneficial photocatalytic properties, and we had already shown that it is possible to deposit metal-sulfides selectively (*e.g.*, PbS, Ag_2_S and Cu_2−*x*_S) on a tip of Cd-chalcogenide NRs,^259^ we combined these two kinds of interfaces into a single multi-component NR, which is based on a CdS (or CdSe-seeded CdS, *i.e.*, CdSe@CdS) NR with two distinct domains on opposite tips: PbS and Pt, forming a SC–SC–M system (PbS–CdS–Pt).^90^ The rationale behind this system is to enhance the charge-carriers’ lifetimes by suppressing recombination due to spatial dissociation, *i.e.*, the electrons are preferably located at the metal tip, while the holes (h^+^) reside on the other end of the complex heterostructure, since PbS acts as a h^+^ sink.

Transient absorption measurements confirm the described kinetic model, but unfortunately, the photocatalytic activity of the PbS–CdS–Pt system does not significantly improve relative to the CdS–Pt one, probably due to the combination of the following: (i) the systems' relatively flat conduction band does not provide sufficient thermodynamic driving force to transfer the electron from the CdS into the Pt tip, while the hole is tightly bound in the PbS, and (ii) electrons are being trapped at the PbS–CdS interface.^90^

Since it is a synthetic challenge to deposit two distinct (single) domains on each NS, Alivisatos and co-workers used a modified approach: they have synthesized a CdSe–Ru dimer as the seed for CdS NR growth. The CdS was successfully grown from the CdSe seed, which resulted in a CdSe@CdS NR with a Ru domain on the surface of the NR (the alkyl chain-length of the employed phosphonic acid could influence the seed position in the CdS). As a final step, a Pt tip was deposited on the NR, resulting in a Ru/CdSe@CdS/Pt HNS.^260^

Schick *et al.* have adapted an encapsulation procedure of MnO in silica to selectively encapsulate only the MnO domain of an Au–MnO heterodimer using a reverse microemulsion technique.^261^ This can be considered a multi-domain formation (a heterodimer) followed by a core–shell formation. The advantage of this method is that the encapsulation does not work on the metallic domain, and leaves it free for further functionalization with thiol molecules, which can allow dispersibility control and facilitate possible biocompatibility-requiring applications such as targeted drug delivery or imaging techniques taking advantage of the simultaneous optical and magnetic responses.^261^

Mirkin and co-workers have published a detailed analysis of palladium–tin alloys showing the possible multiple-domain HNSs with several metals (Ag, Au, Co, Cu) and their bimetallic versions and how they could be spatially arranged up to tetraphase nanoparticles containing six elements.^262^

Significant progress has been achieved by the Schaak group which has used heterodimers (as shown in Fig. 10) as the precursors for development of a wide array of complex inorganic nanostructures with a consecutive-step chemical approach (‘total-synthesis framework’).^51,253,254^ A Pt NP can serve as a nucleation site for Fe_3_O_4_*via* thermal decomposition of Fe(CO)_5_ in the presence of Pt seeds, for example, forming a Pt–Fe_3_O_4_ heterodimer. Then, another metal NP can be selectively placed on the Pt at an opposite direction to the Fe_3_O_4_ domain, resulting in an M–Pt–Fe_3_O_4_ (M = Au, Ag, Ni, Pd) structure.^50^ The chemical selectivity is interesting as the M does not nucleate on the iron oxide, nor at the Pt–Fe_3_O_4_ interface. This selectivity is explained by electron-transfer to the Pt domain, which then facilitates the reduction of the M domain on its surface. A third interface was also added to this system, forming a linear chain of four components, that is an M_*x*_S–Au–Pt–Fe_3_O_4_ heterotetramer, with selective formation of a metal-sulfide (in this report Cu_2−*x*_S and PbS) on the terminal gold.^50^ The key factor to the reported selectivity of the metal-sulfide to the gold is a kinetic competition between reaction of the Cu(i) precursor with the S and the adsorption of S to the HNS. Since sulfur is adsorbed faster on the gold, this is the site where it further reacts to form the metal-sulfide.^50^ A variation of this approach was used by the same group to grow several metal-nitrides on noble-metal seeds; specifically, a three component system was investigated: Cu_3_PdN nucleating on Pt nanocubes–Fe_3_O_4_ dimers.^263^ In this case, the nitride formed preferentially on the corners of the cubic Pt domain while using spherical seeds of Au or Pt results in a core@shell structure. Shi *et al.* have shown already in 2006 that using nucleation of a semiconductor PbE (E = S, Se) on a metal can be used to form a ternary hybrid Fe_3_O_4_–Au–PbE.^264^ The lead chalcogenide nucleates on the available gold domain, which under certain conditions resulted in the formation of PbS NRs as the third component.

The previously described approach is based on successive nucleation events. An alternative mechanism was also reported by Schaak and co-workers, who have used supersaturation and precipitation to transform heterodimers into heterotrimers (in this case, Au–Fe_3_O_4_ into Au–Ge–Fe_3_O_4_).^265^ In this pathway, the metal domain of a heterodimer acts as the center of precipitation for a third material, once it has been supersaturated. This method is less versatile than the ‘regular’ heterogeneous nucleation but has the benefit of minimizing the limitations of surface phenomena and allows the insertion of a distinct metal domain between the metal catalyst and its semiconductor partner in the parent dimer.

**Fig. 11:**
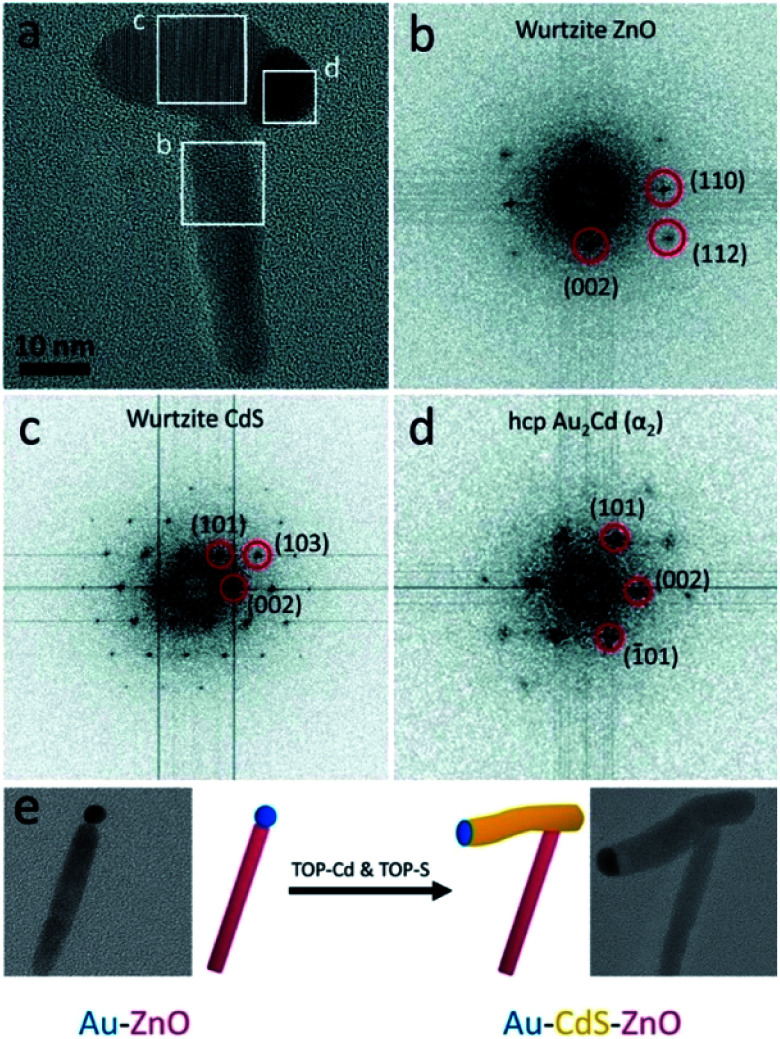
Ternary hybrid system using an SLS mechanism. (a) High-resolution TEM image of Au–CdS–ZnO with marked areas for fast-Fourier transform (FFT) analysis: (b) from the ZnO, (c) from the CdS, (d) from the Au–Cd. (e) Synthetic scheme and TEM images: Au–ZnO NR formed using the seeded growth approach is reacted with Cd- and S-complexes to insert a CdS domain. Reproduced from ref. 266 with permission from the Royal Society of Chemistry, copyright 2017.

Our group has successfully used a related approach to insert a metal-sulfide semiconductor (*i.e.*, CdS) between a preformed ZnO NR–Au tip. As shown in Fig. 11, the CdS precipitates out of the metallic Au–Cd core, pushing the ZnO NR during the synthesis.^266^ We proposed that this insertion mechanism is a form of an SLS growth as the gold tip is alloyed with Cd at the reaction temperature (320 °C), and it serves as the catalyst for CdS precipitation.

An additional option to obtain a complex heterojunction-containing colloidal structure is attachment of different structures in the system as discussed in the review by Buck and Schaak,^51^ followed by fusion. This is usually achieved by controlled elimination of stabilizing agents, which favors controlled aggregation, for example using iodine to ‘weld’ different Au-(multiple) tipped nanostructures as reported by Manna and co-workers.^267^

The ability to form three-component systems can also be used as an intermediate synthetic pathway to achieve two-component products, where the third component acts as a protective layer—to be removed during synthesis. This was reported using a polymer by Xia and co-workers for formation of bimetallic Au–M (M = Ag, Pd, Pt) NPs,^268^ and also in an all-inorganic system by Chen and co-workers, where a silica is protecting a gold core, *i.e.*, an Au–SiO_2_ dumbbell is reacted with another metal, which cannot conformally coat the Au-core due to the existence of the SiO_2_ mask.^269^

This allowed formation of Pd–Au and Pt–Au dimers, as well as Pt–Pd–Au trimers, where dendritic Pt domains were deposited on the Pd, before removal of the protective silica layer from the terminal gold NP. Silica is popular as a hard template that can be removed, as demonstrated in the formation of Pt–Fe_3_O_4_ HNS encapsulated in N-doped carbon hollow spheres by embedding the dumbbell in silica and polydopamine, which after carbonization and silica removal resulted in a yolk–shell morphology.^270^ Naya *et al.* have used ZnO, which dissolves under mild conditions to form a ‘nanoegg’ shape, of half-cut Au(core)–CdS(shell).^271^

### 3.2 Complex (higher-order) systems

**Fig. 12:**
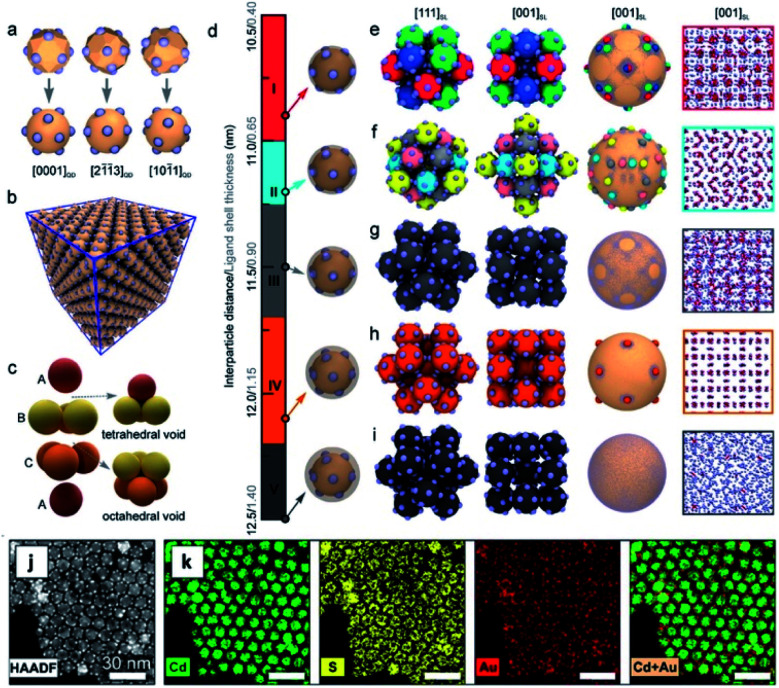
Self-assembly into superlattices from patchy quantum dot–gold hetero-structural nanocrystals without inherent anisotropy. The top part shows simulation of gold NPs on different facets of wurtzite QD hosts with varying interparticle distance adjusted by the ligand shell (surfactant chain-length) and the resulting unit cells: (a) satellite configurations of polyhedral and spherical models, (b) packing into a superlattice with a face-centered cubic (fcc) arrangement of individual patchy NCs, (c) illustration of the voids formed within the fcc lattice, (d) illustration of the five different orientational orders observed in the MD simulation, where the semitransparent gray highlight presents the length of the passivating ligand shell, (e–i) the different orientational order of the structures presenting different projections and a snapshot (right column) highlighting the contacts in red. The bottom part shows experimental high-angle annular dark-field scanning TEM (HAADF STEM) image (j) and the corresponding EDS elemental mapping of CdS polyhedra (9.7 ± 0.5 nm)/Au-satellites (2.2 ± 0.3 nm) HNSs after self-assembly (k), where Cd, S and Au are presented in green, yellow and red, respectively, and the rightmost frame shows the overlay of Cd and Au. Adapted from ref. 39 with permission from the American Chemical Society, copyright 2019.

In addition to increasing the complexity (*e.g.*, morphology, number of components and functionalities) of a single colloidal nanostructure, the different domains constituting an ensemble of HNSs can be used to form complex high order systems such as 3D superlattices and other arrangements on substrates. Much progress has been achieved in using self-assembly for these purposes. For example, Chen and co-workers could successfully demonstrate it using pressure-induced interparticle fusion^272^ and more recently^39^ using patchy Au QDs coupled to interparticle distance control *via* variation of the passivating ligand shell to self-assemble them into different superlattices using NPs without an inherent anisotropy (Fig. 12). The same group also reported how patchy gold domains on CdS–Au_2_S Janus NPs migrate and coalesce to form CdS–AuS–Au heterotrimers upon pressure-treatment.^273^

**Fig. 13:**
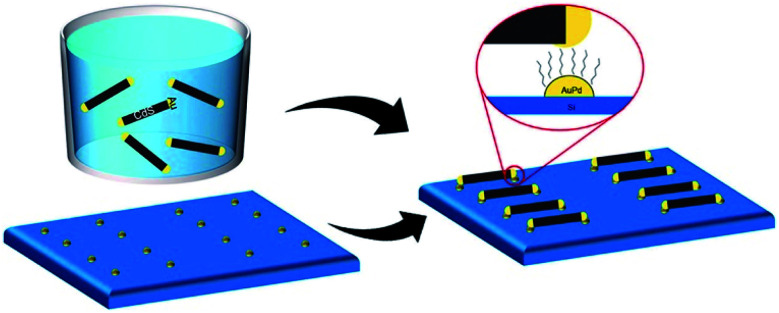
CdS–Au nano-dumbbells self-assemble on pre-fabricated AuPd NPs on a Si substrate (‘nanolithographic docking’).^274^ Reproduced with permission from the American Chemical Society, copyright 2018.

Integration into 2D predetermined patterns can also be achieved using the attraction of metal domains of the HNS to metal anchor points on a substrate (Fig. 13). Such integration was exemplified with Au/CdS NRs/Au nano-dumbbells forming ‘nanolithographic docking’ using the chemical affinity of dithiol molecules to AuPd nanodots on a Si substrate and the Au domains of the HNS.^274^

## 4. Properties and applications

To utilize the unique properties of HNSs in many applications they must be connected to an external circuit when the electric response is required either as an input or an output, including sensing, photovoltaics, (photo)electrocatalysis and so forth. Since an electric signal is transported *via* metallic connectors, there are three common approaches to connect an HNS to an external electrode: (i) direct synthesis of the SC part on a metallic substrate as was discussed with the nickel foam examples in Section 2.2; (ii) deposition of a metal over a SC structure—a possible configuration for photovoltaic devices, where an active layer of semiconductor(s) is sandwiched between a transparent conductive oxide and metal electrodes;^275^ (iii) using the metal part of the HNS itself to serve as the anchoring point to an external macroscale measuring system, for example by connecting a metal to a nanoscale p–n junction. To achieve the latter, lithographic techniques as demonstrated in a recent report by Duan and co-workers^186^ are most common. As these three approaches are less suitable for colloidal HNSs they are mostly outside the scope of this review. Instead, we focus in this section on the utilization of the phenomena arising from the formation of colloidal HNSs in dispersion for (photo)catalytic, photoelectronic and some biomedical applications. Of note is that the solid-state Photovoltaic devices section (Section 4.3) deals with deposited HNS dispersions, which constitute the photoactive layer itself.

For practical utilization of HNSs, the source of the favorable novel properties must be identified. Coupling a metal to a semiconductor can be beneficial for catalysis (Section 4.1) and can improve the light absorption through a plasmonic mechanism or improve the generated charge-carrier dissociation kinetics from the semiconductor—both mechanisms are elaborated in the photocatalysis subsection (Section 4.2).

The formation of a M/SC interface in a colloidal HNS solution also increases the conductance of individual NPs, as exemplified for CdSe NR/Au.^276^ Such phenomena can be used for characterization of multicomponent SC nanostructures using variants of probe microscopy^277^ on the one hand, and for providing a high-chemical yield method to connect metallic domain(s) on various NPs for further integration^276^ on the other hand. The latter is an alternative to the more traditional metal deposition through nanolithography, for integration into nanoelectronics as was demonstrated by the Alivisatos group.^278^ The exact mechanism of conductance enhancement in HNSs is still not fully understood and may change from system to system. Possible contributions (sometimes parallel) may stem from a lower Schottky barrier (shown in single NP measurements^276^ and CdSe/Au nano-dumbbells, which form linked networks),^279^ percolation of metallic domains, and different M–SC electronic coupling phenomena (including photoconduction in Pt–CdSe monolayers^280^) as discussed by Mahler *et al.*, who conclude that the most likely explanation in the case of CdSe NPLs/Au is tunneling events between metal tips with little contribution (if any) from electronic states of the SC.^70^

### 4.1 Catalysis

An interesting example of interface engineering has been recently shown by Zhu *et al.*, who have used galvanic replacement to partially exchange Cu NPs into Cu@Ag NPs alongside partial oxidation of the Cu to Cu_2_O.^140^ Control over the oxidation allowed formation of a thin layer (3–6 atomic layers) of Cu_2_O, which induced compressive strain at the NPs' surface, which was demonstrated as beneficial for the catalytic transformation of aniline into azobenzene (oxidation). Another common model reaction to test SC/M systems is the (photo)reduction of 4-nitrophenol (4-NP) to 4-aminophenol in the presence of sodium borohydride (NaBH_4_),^117^ which was recently shown to take place on the catalyst's surface—adsorbed 4-NP molecules are reduced using electrons from the catalyst and protons coming from the protic solvent (water, alcohol, *etc.* but not directly from BH_4_^−^).^281^

The oxygen reduction reaction (ORR) is a reaction in the field of batteries (Li–O_2_ batteries, for example)^282^ and fuel cells,^283^ where an electrocatalyst (most commonly a precious metal) is responsible for the transformation of O_2_ into H_2_O (in the case of Pt(100) the ORR is performed *via* the oxygen dissociation mechanism).^284^ Using the interaction between the metal-oxide support and the metal,^285–288^ the energetics of the catalyst can be improved. Meng *et al.* have epitaxially grown Pt on single-crystal CuO NRs for this purpose.^285^ They have loaded a carbon cloth with fcc CoO NRs with pyramidal facets, which served as the nucleation centers for epitaxially grown fcc Pt NPs (∼7% lattice mismatch) using magnetron sputtering followed by annealing to achieve crystallization.

The reported Pt/CoO configuration results in electron donation from the oxide to the metal, which tunes the d-band structure of Pt in the hybrid system downwards, and results in favorable intermediate adsorption.^289^ This is an elegant demonstration of the electronic charge transfer into a metallic catalyst, which improves its catalytic efficiency. As pointed out by Wang *et al.*, this is only one contribution to ORR activity and other effects such as particle size, crystal facets and lattice strain have a critical impact, which is usually hard to decouple from the charge transfer effect in hybrid structures (either M–support or M–M).^289^ Further advancement is expected when the metal-oxide support is doped, which can improve its conductivity, stability of the active electrochemical and specific surface areas, as well as tuning of the energy alignment with the metal catalyst.^283^

The hydrogen evolution reaction (HER) and related reactions are very common using a metallic catalyst coupled to a transition-metal chalcogenide or pnictide as was discussed in Section 2.2.^290^ As a rule-of-thumb, the most robust systems are expected to be grown directly on a conductive substrate that acts as the electrode, for example, Co/Co_3_O_4_ core/shell 3D structure over NF.^232^

Another electrocatalytic reaction of interest is the electrooxidation of alcohols. This reaction is usually performed using metal catalysts such as Pd or Pt and supported on metal oxides such as CeO_2_,^104,291^ TiO_2_,^291,292^ SnO_2_,^291^ Ni(OH)_2_ (ref. 293) and MoO_*x*_^294^ in alkaline media with a strong influence of the support on the selectivity of the process.^291^ The support–metal interaction is just another example of the novel properties arising in HNSs, which include charge transfer from the metal and improved oxygen binding of surface metal centers in the metal-oxide (surface oxygen vacancies); in electrooxidation these bound oxygen-containing species can be especially beneficial for the removal of poisoning intermediates from the metal surface.^104^ Other variants include PtAu/Bi_2_O_3_,^295^ Pt/CeO_2_/PANI (the polyaniline (PANI) polymer allowing 1D nanotube morphology)^296^ and Pd coupled to Co-doped CeO_2_ dots, which result in increased poisoning resistance.^297^ TMPs are also frequently used in conjunction with a carbon support, *e.g.*, Pt over Co_2_P,^298^ PtP over NiCo_2_P_*x*_,^299^ and Pd/CoP.^300^ As a model electrocatalytic system, our group demonstrated EtOH oxidation using an insulating 3D scaffold of CaCO_3_ from a marine origin (*Sorites*), coated with cobalt and gold.^301^

### 4.2 Photocatalysis

Photocatalysis is probably the most common application of M/SC HNSs. The first step in a photocatalytic reaction is optical absorption, which is a light–matter interaction heavily influenced by the coupling extent between quantum states of the HNS's components. There is a strong influence of the material and shape of the SC (see for example, Kuno and co-workers),^302,303^ and it may be further altered after the formation of the M–SC heterojunction(s).

As discussed by Sönnichsen and co-workers, CdS–Au matchsticks retain similar optical properties, while CdSe–Au dumbbells exhibit significant changes relative to their pristine SC NC.^304^ The strong M–SC interaction alongside spectral overlap between the CdSe excitonic band edge and the plasmonic Au resonance frequency leads to smearing of the ‘plasmonic’ absorption peak and complicates theoretical calculations. This observation leads the discussion to the second step in photocatalysis—generation of charge carriers and their migration. The basic model is that a semiconductor absorbs at its band edge (*i.e.*, *hν* ≥ *E*_g_), which leads to an electron excitation from the valence band (VB) to the conduction band (CB), where a hole is thus formed; a metal has a plasma frequency, where it also absorbs light, which if intense enough at the relevant wavelength causes excitation of free electrons, which in turn can participate in some kind of energy transfer.

Pradhan and co-workers have shown how the incorporation of Sb ions into a bismuth sulfide NR matrix improves light absorption of this non-heavy metal SC. These NRs were grown on gold seeds, forming a photocatalytically active Au–Bi_2−*x*_Sb_*x*_S_3_ hybrid system.^305^ Two important features of this work are the shortening of the elongated Bi_2_S_3_ upon introduction of Sb with a single encapsulated Au NP per NR, and the addition of LSPR to the SC. It is important to mention that metal-sulfides are known for their strong localized surface plasmon resonance phenomena (due to sulfur vacancies). The energy of the resonance can be tuned by variation of the material as demonstrated by Gabka *et al.*, who have synthesized Cu–Fe–S nanocrystals,^306^ or by formation of a heterostructure (variable ZnS domain length) to tune the nanoantenna (Cu_1.96_S) as was shown by Wang and co-workers.^307^ These materials allow a rational planning of hybrid materials, where the plasmonic response comes from the SC and not from the metal.

The third photocatalytic step is the charge and energy transfer phenomena, usually to the interfaces—the solid heterojunctions and to the surfaces of the HNS, where they meet the molecules relevant to the last catalytic reaction (surface bound ligands, solvent molecules and any other adsorbed molecule, including reactants). At this stage, the quality of the interface, the materials, and their morphology play a significant role (*e.g.*, affecting the kinetics of electron transfer), as will be discussed for the relevant applications. For example, Yu *et al.* have shown a difference between electrons excited to the band edge (‘cold electrons’) and high-energy electrons (‘hot electrons’) in CdSe NRs. Comparing CdSe NR/Au and CdSe NR/Pt HNSs showed that hot electron (e^−^) injection is faster for the Au-tipped HNS while the cold e^−^ injection yield is higher in the Pt-tipped one.^308^

To describe the role of the M/SC interfaces we deal with two main cases: (i) the metal serves as an antenna for light-harvesting through a plasmonic mechanism, and this energy can be transferred to the SC, thus enhancing the photocatalytic activity;^125^ (ii) the semiconductor absorbs the incident radiation, and the metal acts as a sink for the electrons, which can then perform a reduction reaction, while the holes remaining in the SC can perform an oxidation reaction. As in all heterogeneous catalytic reactions, the metal surface also provides adsorption sites.

The plasmonic mechanism is very appealing since this mechanism results in an experimentally improved absorption cross-section of the metallic domain relative to the purely geometric one^309^ (which is relatively low for small colloidal NPs). The metal can thus photo-enhance catalytic reactions,^310^ with an adjacent wide bandgap SC or insulator (a stable oxide) acting as a support. For example, the Linic group has shown the photocatalytic activity of alumina-supported silver nanostructures towards oxidation reactions.^311^ They have ascribed the plasmonic effect manifestation in the form of an electron transfer to antibonding orbitals of oxygen in the adsorbed reactants. Many others have used a variety of metal–metal-oxide combinations; some recent examples include Ag^312^ or Rh^313^ nanocubes supported on α-Al_2_O_3_, different Au NPs supported or coated on TiO_2_,^314,315^ SiO_2_,^316,317^ or both (*i.e.*, Au@SiO_2_@TiO_2_),^318^ as well as multi-metal combinations as Al antenna–Ir NPs on an Al_2_O_3_ support^319^ for photocatalysis or for studying the interaction arising at the M/SC interface.^320^

Of note are the studies of Li, Wei and co-workers, who used plasmon-enhanced Au core(s)@MoS_2_ sphere shell HNSs for photocatalytic hydrogen production with stable high rates.^321,322^ In one configuration, the proximity between gold multimers (spacing of 5–10 nm) allowed enhancement of the absorption cross-section and an additional absorption frequency at *ca.* 630 nm appeared due to in-phase coupling in a single-material gold multimer system. A subsequent MoS_2_ shell formation results in an HNS with strong absorption both at the characteristic Au response (∼640 nm) and at the MoS_2_*E*_g_ (∼510 nm). The multimer hybrid thus exhibits both an improved absorption in the visible range and a higher energy transfer rate relative to single Au@MoS_2_, improving photocatalytic H_2_ production. The relative high stability of this colloidal system stems from the strength of the Au–S bond in the anchoring of MoS_2_ to gold and provides an additional photocatalytic advantage.^321^ The other system with high stability is the ternary HNS formed by reacting Au@MoS_2_ hydrothermally with a zinc source and an amine to form Au@MoS_2_–ZnO.^322^ The latter system benefits from the mechanical strength of the NRs in the composite (about 6–10 rods per sphere) and higher specific surface area, but more importantly, the introduction of ZnO alters the electronic structure resulting in improved light harvesting in the 350–700 nm range: a new absorbance intensity is measured in the UV (350–400 nm) and the authors ascribe the red shift of the typical MoS_2_ absorption at *ca.* 600 nm to 650 nm to the surface plasmon local field effect. Furthermore, ZnO, a known electron conductor, facilitates spatial charge separation, and in part is responsible for the reduced photocorrosion.^322^ For further plasmon-enhanced applications we refer the reader to a review from the Wei group regarding various metal–2D hybrids (graphene, MX_2_, BN, *etc.*).^191^

**Fig. 14:**
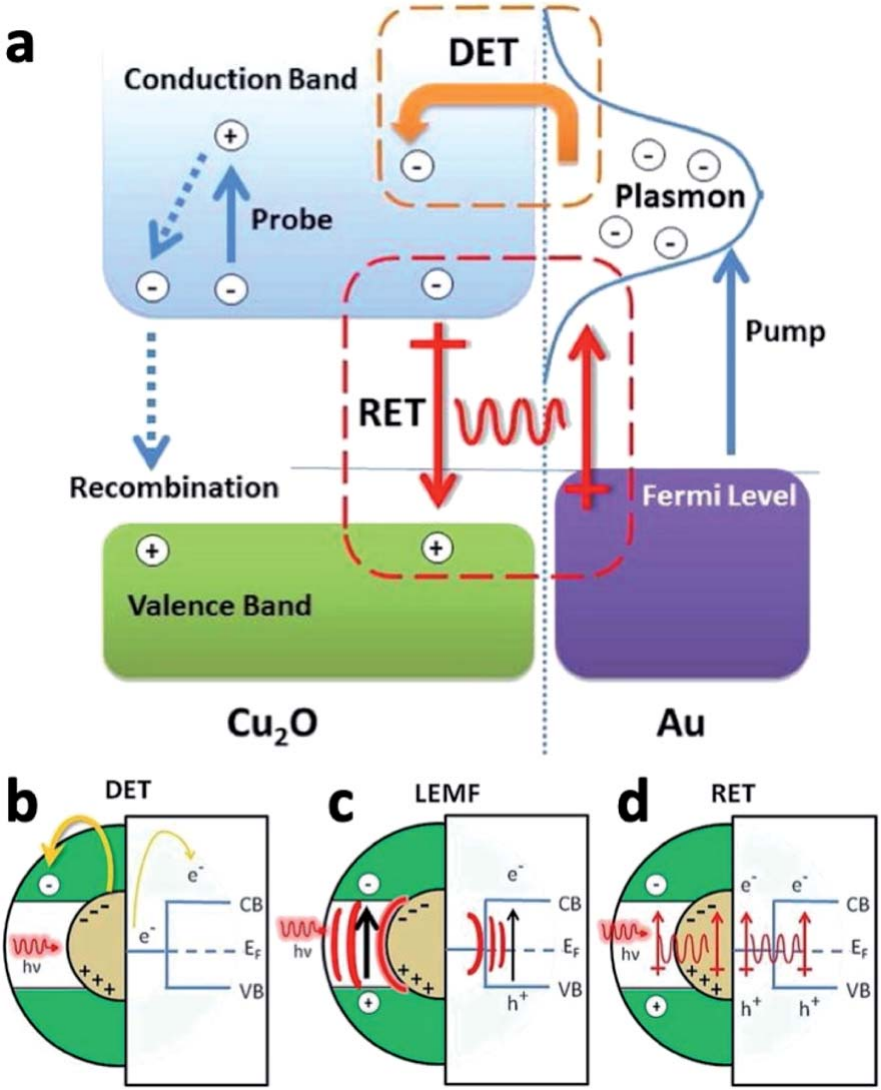
Various possible energy-transfer and separation processes responsible for plasmon-enhanced photocatalytic activity of Cu_2_O/Au-based HNS.^125^ (a) Scheme of energy absorption at the metal (pump) and the subsequent energy-transfer processes: (b–d) illustration of the metal core (Au) and the SC shell (Cu_2_O) energy positions (right) and processes (left): (b) direct energy transfer (DET), where LSPR ‘hot’ electrons are transferred to the SC's CB; (c) local electromagnetic field enhancement (LEMF), where the e^−^–h^+^ charge-separation in the SC is radiatively aided by the metal excitation; (d) resonant energy transfer (RET), where the LSPR dipole induces e^−^–h^+^ pair separation in the SC *via* a non-radiative mechanism that relaxes the localized surface plasmon dipole. Adapted with permission from the American Chemical Society, copyright 2012.

To explain the photocatalytic enhancement of the HNS, the commonly suggested modes of energy transfer are as follows: (i) the metallic NP, which absorbs the radiation performs a direct energy transfer between the LSPR and the coupled electronic state of a damping surface-attached chemical species (*i.e.*, catalysis on the metal's surface), or (ii) indirect energy transfer, where the ‘hot’ carrier from the plasmonic structure is an intermediate agent that allows the dephasing of the surface plasmon and transfers the excess energy either as an electric charge or as vibrational energy to the interface at the surface.^323^Fig. 14 further elaborates the possible energy transfer mechanisms from the metal to the SC, based on the work of Cushing *et al.* on a silica-passivated M@SC shell (Au@SiO_2_@Cu_2_O).^125^ As they point out, there is still no consensus regarding which mechanism is at play for the reported systems, and novel probing techniques are constantly recruited for this task.^324–327^

One of the consequences of the plasmonic response is local heat generation through electron–phonon scattering,^318^ which has made a distinct contribution to successful photocatalytic CO_2_ methanation of Rh/TiO_2_, for example.^318^ It has been suggested recently that the catalytic activity of the generated ‘hot-electrons’, *i.e.*, non-thermal charge carriers in the metallic nanoparticles (which are responsible for the photocatalytic enhancement reported for Cu–Ru NPs^328^ and others^312,315,317^) disregards the local heating effect of the plasmonic structures, and that the measured enhanced photocatalytic effects should be ascribed, *in lieu*, to thermal effects (well-described using a simple Arrhenius model).^329–332^

This intriguing explanation requires significant improvement of experimental apparatus and procedures both to perform correct control experiments and to allow the measurement of real local temperatures. It paves the way to further improvements by taking into account the resulting thermal gradients^333^ as well as rational design of HNSs that can sustain enhanced heat generation, for example by improving the heat conductance of the supporting oxide.^330^

The metal as an electron sink is the other common way to imbue enhanced photocatalytic properties to an absorbing SC nanostructure. The two most commonly reported photocatalytic applications are: (i) light-induced heterogeneous catalysis, mostly performed in a solution, where the colloidal HNSs are suspended, and (ii) photoelectrochemical cell (PEC) setups, where the HNSs are deposited on a photoanode or a photocathode. Colloidal-phase photocatalytic reactions are mainly oxidation or reduction reactions (possibly with radical intermediates) taking place in aqueous or organic media. Though organic-phase photocatalytic oxidation reactions exist (*e.g.*, 2-mercaptobenzothiazole (MBT) oxidation demonstrated using cesium-lead-halide perovskite QDs in hexane,^334^ benzyl alcohol oxidation using titania and titania–iron oxide^335^ or using CdS and/or TiO_2_ in MeCN (acetonitrile) and or MeCN–trifluorotoluene mixtures^336^ and alcohol dehydrogenation in MeCN^67^), they are less common for M/SC hybrids as the metal serves as the electron sink. Therefore, when a SC/M hybrid is used, the electrons from the sink are consumed for the formation of radical species such as ˙O_2_ from dioxygen, which are consumed during the oxidation reaction.^337^

A report by Wang *et al.* describes synthesis and use of a hybrid Pd/ZnIn_2_S_4_ catalyst for alkylation of amines and ketones in alcohols. The organic reaction mechanism involves the dehydrogenation of alcohol to aldehyde, followed by condensation of the aldehyde and a nucleophile (amines and ketones) and then hydrogenation forming the final alkylated product. The semiconductor part photocatalyzes the dehydrogenation (the suitable valence band position slows controllable oxidation), while the Pd catalyzes the hydrogenation of the intermediate at the final step.^338^

**Fig. 15:**
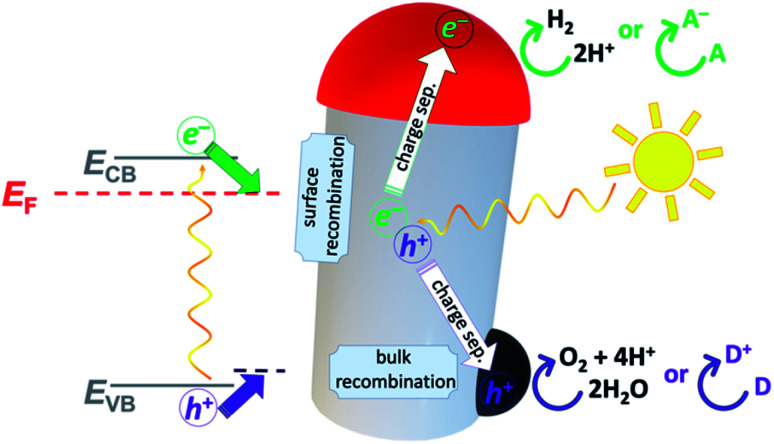
Schematic charge separation diagram (spatial paths and energy bands) on an HNS, illustrated on a SC nanorod with an M tip (reduction catalyst) and an additional oxidation cocatalyst. First, a semiconductor NR (gray) is excited with sufficient energy to excite an electron from the valence to the conduction band; the desired action is spatial charge separation, where the electron is drawn towards the metal tip (red), which serves as an electron sink at lower energy (the Fermi level, *E*_F_). From the metal, the electron can perform reduction of any adsorbed acceptor (A → A^−^), which in the case of water splitting are protons being reduced to hydrogen (*i.e.*, the HER); in dye degradation experiments the e^−^ can reduce the dye or form intermediate ˙O_2_^−^ species in the presence of oxygen, which in turn form hydrogen peroxide, and finally destructive hydroxyl radicals. While this is occurring, the h^+^ oxidizes an adsorbed donor molecule (D → D^+^), which in the case of water splitting is water to oxygen (*i.e.*, the OER) and in the case of dye degradation a possible reaction path is hydroxide (water) oxidation to hydroxyl radicals (and H^+^), which in turn mineralize the dye. The competing processes are bulk (volume) and surface recombination, occurring throughout the SC or at its surface (*e.g.*, due to some passivation defect), respectively. To minimize recombination, the charge carrier kinetics is crucial—for example, while no donor accepts the hole, it serves as a coulombic attraction center for electrons. When the h^+^ removal is a sluggish process, as is the case in the OER, a possible solution is the addition of a cocatalyst (represented in dark gray), which catalyzes water oxidation, thus removing the h^+^ from the HNS and suppresses recombination.

Probably the most common aqueous-phase photocatalytic reactions are organic pollutant dye degradation and variants of water-splitting (the HER and/or the OER)—both requiring a ligand-exchange procedure, if the HNSs are initially synthesized in an organic phase, to allow their dispersibility. The spatial charge separation pathways and the corresponding energy band diagram are presented in Fig. 15.

Examples of dye decoloration and degradation (mineralization) include methylene blue (MB) using MUA (mercaptoundecanoic acid)-capped Pt/CdS/PbS NRs^90^ and CZTS NRs/Au,^86^ toluidine blue using ZnO of various shapes/Au^63,167^ and ZnO/Au/Pt,^64^ rhodamine B (RhB) using calcined Au/ZnO nanopyramids^339^ or Au/Ni/ZnO flower HNSs,^168^ rhodamine 6G (Rh6G) using ultrasonicated tip-attached Ag/ZnO nanopyramids,^169^ azo-dyes such as methyl orange (MO) using ultrasonicated Au/ZnO nanopyramids^60^ or Au–Cu_2_O^120^ and orange G using Ag/ZnO hollow spheres.^340^ In the last example, the authors verified that the enhanced degradation stems not only from enhanced charge separation but also from the increased concentration of hydroxyl radicals (˙OH), formed from surface-attached hydroxyls. 4-NP reduction was demonstrated with triangular- and prism-shaped Au domains/ZnO HNSs, prepared in an aqueous phase.^341^ Epitaxial Au–triangular ZnO NPs (and Pt–Au–ZnO) were used to study the charge accumulation and discharge using degradation of toluidine blue, which allowed monitoring not only degradation (photoreduction) but also measuring on/off characteristics due to its ability to reoxidize.^64^

As these examples show, a metal domain is beneficial for dye degradation since it attracts photoexcited electrons, and then these electrons may generate hydroxyl and superoxide radicals, which then attack the dyes. To this end, a good practice is to measure not only photodegradation kinetics but also to detect the radicals using methods such as electron paramagnetic resonance (EPR) and a spin trapping agent such as 5,5-dimethyl-1-pyrroline-*N*-oxide (DMPO).^168^ It is important to stress that especially in the case of the common MB dye, decoloration can result from reversible photoreduction to the leucomethylene blue form,^86,342^ which is different from complete degradation (*via* a photooxidative path).^343^ MB can also be degraded from an alcoholic solution, where a catalytic amount can be dispersed even in an organic solvent (*e.g.*, Au–Cu_2_S in hexane degrades MB in hexane).^146^ Related reactions, which provide further physical insight, are site-specific dye transformations. For example, Ha *et al.* have used an Au–CdS NR HNS to transform an amplex red dye (non-fluorescent) into resorufin (fluorescent) in the presence of H_2_O_2_. This work demonstrated the ability to distinguish between the charge carriers (h^+^ and e^−^), which are generated from distinct excitations, namely the gold metal domain, which is excited at its plasmonic resonance wavelength and the CdS SC, which is excited at its shorter-wavelength first excitonic band edge.^344^ Since the product is fluorescent, high-resolution localized imaging became possible. In a related report, fluorescent ATTO dyes were used to probe energy transfer and charge separation mechanisms.^345^

**Fig. 16:**
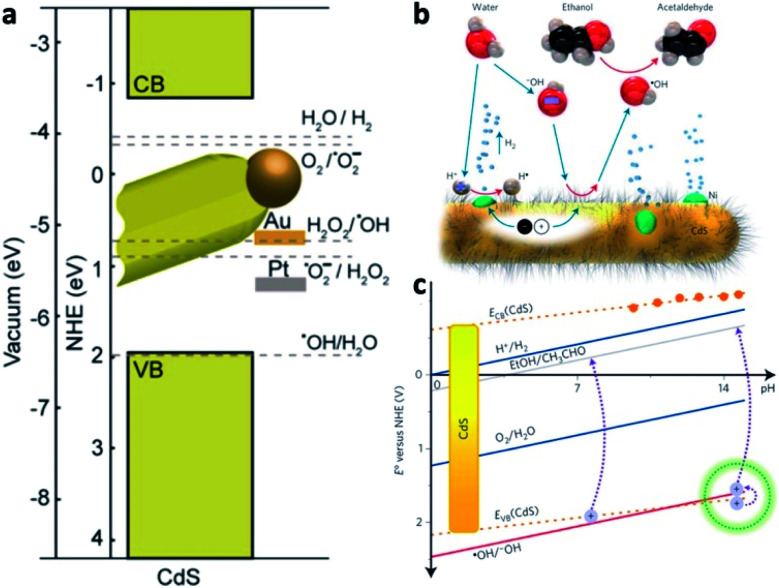
Experimental energy band alignments for photocatalytic applications of CdS NR/M HNSs. (a) CdS NR (CB and VB) with either Au or Pt tips (*E*_F_) and the relevant HER, OER and intermediate radical energy positions on the electrochemical normal hydrogen electrode (NHE) and absolute vacuum scales. Reproduced with permission from ref. 348, Wiley-VCH Verlag GmbH & Co., copyright 2018. (b) Schematic reaction of the CdS NR/Ni photocatalyst producing H_2_(g) (HER) and oxidizing EtOH using a hydroxide intermediate, (c) the corresponding energy scale (*y* axis) of these reactions as a function of pH (*x* axis); reproduced with permission from ref. 69, Springer Nature publishing group, copyright 2014.

Dye degradation experiments are a facile method to test for photocatalytic activity, but much effort has been invested in using colloidal nanostructures of different compositions for direct utilization for water-splitting (specifically, the HER). Noble-metal/stable wide-bandgap oxides have been tested for this purpose for more than two decades (photo-deposited Pt on TiO_2_, for example, for direct photolysis or with alcohol hole-scavengers,^346^ as well as other electron donors such as oxalic acid).^347^ A thorough investigation by Banin and co-workers has shown the effect of metal type at the tip of CdS NRs on the formation of reactive oxygen species (ROS), which are the intermediates of the hydrogen evolution reaction (see Fig. 16a).^348^ They have synthesized single-tipped CdS NRs (48 ± 5 nm × 3.3 ± 0.5 nm) with either gold (2.5 ± 0.6 nm) or platinum (1.9 ± 0.5 nm) tips. Surprisingly they have found that though Pt is considered the ‘better’ H_2_(g) production catalyst, Au-tips have higher photocatalytic efficiencies towards H_2_O_2_ and ˙OH generation.

Non-noble metal decorated HNSs were reported, *e.g.*, NiCd/CdS^154^ or Ni/CdS^69^ NRs. In the latter colloidal system, the hole scavenger is ethanol being oxidized to acetaldehyde alongside a reductive HER (Fig. 16b and c). In this report, hydroxides on the surface of the CdS NRs act as a ‘shuttle’—they take the hole and oxidize the EtOH, with significantly higher HER rates with increasing pH.^69^

Another aspect to the role of the metal as an electron sink is that it might reduce electron–hole recombination, thus facilitating oxidation reactions, where the hole ‘attacks’ the oxidized species on the SC's surface. Such an example is the oxidation of benzyl alcohol by a TiO_2_/Pd hybrid in water.^337^

In the well-studied CdS NR/Pt system, Lian and co-workers found that the long-lived charge-separated state (∼1.2 ± 0.5 μs) stems from the spatial separation between the electron in the Pt tip and the hole trapped in the CdS rod.^349^

Since a noble metal generally allows fast electron transfer kinetics (SC to M charge transfer is fast^349–351^ and Pt, for example, has low overpotential for oxonium reduction), the hole transfer for water oxidation in water splitting reactions is usually the rate determining step. To circumvent this obstacle several approaches have been utilized:

(1) To use a hole scavenger—a molecule in the solution that is an electron donor, D, such as alcohol, SO_3_^2−^, triethanolamine or EDTA^4−^,^352^ thus exploiting only the HER to obtain useful H_2_(g).

(2) To help increase the h^+^ lifetime, the SC part can be a core@shell nanostructure, preferably with the buried core, spatially far from the metal. For example, when a CdSe core is present inside a CdS NR, due to quasi-type II band alignment, the holes might be localized within the CdSe core, while the electron's wavefunction is spread throughout the NR, which allows its injection into a metallic tip. As described by Lian and co-workers, the actual charge dynamics is more complicated with several relevant time constants of the excitonic and charge-separated states.^351^ Two key points from this account are (i) the role of the phase-transfer agent, *i.e.*, MUA or MPA (mercaptopropionic acid) ligand, whose thiol end attaches to allow the HNS's dispersibility in water, acts as an electron donor, and (ii) the charge transfer dynamics changes when the electron donor is changed, thus it is established for this system that the efficiency-limiting step is the hole removal.^351,353^

(3) To deposit an oxidation catalyst on the SC to allow fast oxidation of the donor molecule, usually OH^−^ for the OER in an alkaline environment. Wolff *et al.* have used the very-well established double-sided Pt-tipped CdS NR system (nano-dumbbell) with a Ru-based molecular oxidation catalyst, anchored on the sides of CdS through dithiocarbamate bonds, for efficient water splitting.^204^ From transient absorption measurements, they conclude that the hole transfer to the molecular catalyst (which oxidized the Ru^2+^ center to the Ru^3+^ state) is also very fast, *ca.* 300 fs.^204^ This fast kinetics is achievable by using the dithiocarbamate bonds, unlike similar adsorbed Ru-complexes on CdS NRs.^195^

Amirav and co-workers have synthesized with this goal in mind hybrids of CdS NRs with IrO_2_ (ref. 354) and Ru_*x*_O_*y*_^260^ NPs that showed extended durability under illumination. They are candidates for overall water splitting as demonstrated by Grätzel and co-workers,^355^ since iridium and ruthenium oxides are excellent oxygen evolution cocatalysts with the lowest overpotentials.^356^ Khan *et al.* deposited CoO_*x*_ on CdSe NRs as the oxidation cocatalyst and also synthesized Pt/CdSe NR/PdS dual-cocatalyst HNS for the HER, where the selectively deposited Pt tips catalyze a reduction reaction while PdS NPs decorating the surface catalyze the oxidation one.^357^

Many additional variations to the cadmium chalcogenide NR/M system were attempted to improve the stability and increase catalytic efficiencies—including variation of the NR's dimensions, CdSe@CdS seeded NRs, variation of tip size, number of metal catalysts, composition of the tip (core–shell, multi-domain, alloy bimetallic tips) and so forth.^100,348,351,358–360^

It is important to note that since the reaction conditions and measurement protocols are not standardized, it is hard to compare between different reports, especially since the illumination intensity and wavelength can change the number of photoexcited charge carriers per HNS and influence the charge transfer mechanism. As pointed out by Ben-Shahar *et al.*, this is especially important in photocatalytic reactions involving multi-electron reactions such as water splitting.^361^

As mentioned in the plasmonic mechanism of photocatalysis, careful planning of a HNS can allow harvesting additional energy with the metal: for example, by harnessing the enhanced scattering of gold coupled to CdS, alongside catalytic Pt tips in a triangular Au–Pt–CdS hybrid.^362^ Recently, Kawawaki *et al.* have demonstrated that an additional interfacial layer of Ag_2_S between a CdS NR and an Ag tip increases the plasmonic enhancement towards the HER.^363^ The silver sulfide domain in this Ag–Ag_2_S–CdS HNS serves as a carrier-selective blocking layer, *i.e.*, prevents the electron from migrating away from the CdS towards the metal sink, thus the staggered band alignment between the semiconductors facilitates charge separation (electrons in CdS and holes in Ag_2_S), while maintaining the plasmonic effect. Furthermore, the h^+^ removal from CdS lowers its self-dissolution, one of the main practical problems in using CdS (sometimes referred to as photoetching).

The discussion thus far focused on model contaminant organic dye degradation and water splitting. Similar principles can apply to other photocatalytic systems such as the much sought-after CO_2_ reduction.^364,365^ The basic principle of such solar fuel production systems is that the electron reduces an adsorbed reactant CO_2_ with water to produce some valuable substance, *i.e.*, the CO_2_ is the acceptor molecule (A) in Fig. 15, which is reduced to A^−^, for example CO and CH_4_ on Cu_2_S NR/Pt HNS.^71^ Other reactions include biomass valorization (oxidation to useful chemicals) alongside H_2_ production using CdS nanosheets/Ni,^214^ and even coupling to biological enzymes such as horseradish peroxidase^366^ since M/SC HNSs generate radicals, allowing building a setup for fundamental scientific investigation of a biological ‘machine’. Of note is that photoreduction can be used as a method to probe photogenerated charge distribution, for example resulting in oxidation of Pb^2+^(aq) to PbO_2_ or reduction of CrO_4_^2−^(aq) to Cr_2_O_3_.^367^

Photoelectrochemical cells expand the photocatalytic applicability of HNSs since they allow the application of an external electrical bias. The arising difficulty is the need to deposit (or grow in the first place) the HNS on a conductive electrode, and that mass transport phenomena and charge carrier diffusion lengths within the absorbing (thin) layer become much more significant during the photocatalytic stage. The HNS is deposited on a photoelectrode (usually with a polymeric binder such as PEDOT:PSS or Nafion). For example, Au–Bi_2−*x*_Sb_*x*_S_3_ NRs with LSPR-enhanced absorption were deposited on an indium tin oxide (ITO)-coated glass, which served as the photocathode, after a ligand-exchange procedure to MPA.^305^ In this case, the best results were obtained when a hole-selective transport layer of PEDOT:PSS was deposited between the ITO and the HNS. Similarly, Au/CuInS_2_ discs (Fig. 5g) were used in PEC photoanodes with and without a hole transporting PEDOT:PSS layer.^129^ Another possible deposition method is using a chemical bond. For example, 2-mercaptoglycolic acid-modified CdS NRs (loaded with varying Co_3_O_4_ amounts) are reacted in acidic pH to form an ester bond with the conductive ITO electrode.^221^

As previously mentioned, plasmonic effects can enhance the performance of thin-film devices. For example, a gold underlayer improves the photocatalytic performance of a p-type copper(i) oxide photocathode (electrodeposited Cu_2_O above Au/ITO) through both scattering and resonance mechanisms.^368^ Another benign oxide was used as a photoanode by the Yang group to fabricate an Fe_2_O_3_ (hematite) overlayer above ∼300 nm nanoimprinted Au nanopillars to utilize enhanced light absorption.^369^ Besides serving as a functional solar-fuel production device, PEC can also provide information regarding charge dynamics in an HNS. For example, the thoroughly discussed CdS (or CdSe@CdS) NR/multiple Au domain system was also investigated by Bigall and co-workers *via* linking on ITO with (3-mercaptopropyl)trimethoxysilane (MPTSMS).^370^ One of their findings is that the gold domains allow an improved contact between the NSs and the conductive substrate, and also allow measuring both positive and negative photocurrents at an appropriate bias voltage, a crucial feature for possible PEC sensing applications.

### 4.3 Photovoltaics

Photovoltaic devices, either all-solid-state, or some variation of a dye- or QD-sensitized solar cell (where a liquid electrolyte is present) use semiconductor(s) to harvest solar energy as a first step before the excited charge carriers are separated, and transported to two counter electrodes, thus inducing the photovoltaic energy harvesting. Although a photovoltaic device requires a metal contact, it is not commonly interfaced directly to individual SC nanostructures. The first reports using colloidal SCs as the absorbing material in a polymer matrix such as CdS and CdSe were reported more than two decades ago,^371,372^ and today they commonly exceed 10% photon-to-electron conversion (PCE) efficiencies.^373^ The benefit of using metallic NSs in a polymer blend is their light manipulation contribution, most commonly LSPR (*e.g.*, Au NRs,^374^ Ag nanoparticles,^375^ and Au/Cu_2−*x*_S HNSs, which exhibit a broader scattering range than standard noble metal NPs^376^). We refer the reader for additional light management and manipulation strategies of metallic features such as incorporation into charge-selective layers, reflective surfaces and additional relevant aspects of plasmonics elsewhere.^377–381^

Combining these two approaches, as mentioned above, results in the incorporation of M/SC HNSs in a polymer blend, which increases the absorption of a thin active layer using both the SC property and the plasmonic effect. For example, Au/Cu_2−*x*_S Janus particles with enhanced absorption in the 500–700 nm range were incorporated into an organic solar cell blend (specifically, into a bulk heterojunction cell (BJC), with the HNS being mixed into the P1:PC_71_BM blend, reaching PCE values of almost 5%).^382^

### 4.4 Biomedical applications

The tunable properties of HNSs make them candidates for various biomedical applications ranging from bioimaging to active phototherapeutic materials (a representative example will be the previously discussed ‘classic’ system of CdSe seed@CdS NR/Au NPs).^383^ The main concern regarding nanomaterials and the limiting factor for their application, in general, is their toxicity; much progress is being achieved recently in this respect for all kinds of nanomaterials.^384^ One possible cure is using heavy-metal-free materials, with significant progress achieved, for example, in SC NR-related systems.^385^

**Fig. 17:**
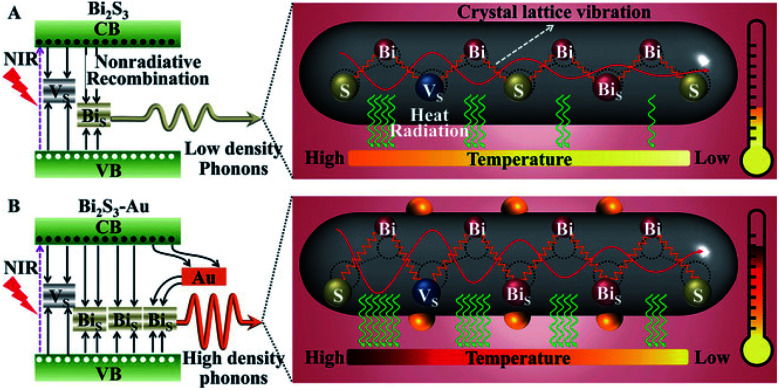
Schematic photothermal mechanism of (A) Bi_2_S_3_ NRs and (B) Bi_2_S_3_/Au HNSs. On the left side an energy diagram shows the SC band edge absorption, vacancy levels and possible gold incorporation are translated into phonons (crystal lattice vibrations, depicted on the right side) that generate the heat. Reproduced from ref. 386 with permission from Wiley-VCH Verlag GmbH & Co., copyright 2018.

Cheng *et al.* have shown a photothermal effect in a Bi_2_S_3_ NR/Au NP system, where bismuth sulfide absorbs a near-infrared (NIR) signal (808 nm laser) and the non-radiative recombination is enhanced *via* the decorating gold NPs, as shown in Fig. 17.^386^ Other examples include SiO_2_/Au/Fe_3_O_4_ multi-shell structures for photothermal treatment of breast cancer cells and magnetic stimuli functionality for MRI.^387^

Related reported systems also include other hybrids with insulators or double hydroxides, such as core–shell Au NRs in Al and Mg hydroxides^388^ and Au NRs partially embedded in silica (Au–SiO_2_ Janus particle with the Au NR partially exposed) that show improved infrared response for photo-triggered drug delivery alongside improved loading.^389^ Another advancement was reported by Jiang and co-workers, who have synthesized a material with a strong response in the second NIR window (they measured light penetration at 1064 nm) using Au/Cu_9_S_5_ HNS, where both the metal and the semiconductor have an LSPR response.^390^ A related spherical core@shell Au@Cu_2−*x*_E (E = S, Se) was demonstrated by Zhu *et al.*, where the coupling of the plasmonic response of the core and the shell was responsible for high extinction coefficient at 808 nm.^391^

Huang and co-workers have used the combination of facet-dependent absorption and a plasmonic response to tune photothermal effects of a simple Au–Cu_2_O system.^113^ Sun *et al.* have recently shown a very high photothermal anticancer activity with sub-stoichiometric tungsten trioxide (WO_2.9_) NRs, which exhibit partial metallic character.^392^

It is envisioned that this kind of systems can be further enhanced by an HNS formation and other modifications such as cation intercalation (*e.g.*, NH_4_^+^).^393^ Biocompatible magnetic domain-containing HNSs are also promising due to their possible magnetic-controllable catch and release, as demonstrated for Au– and FePt–Fe_3_O_4_ heterodimers,^394^ and multimodal imaging using M/Fe_3_O_4_.^108^

## 5. Perspective

This review presents the synthesis and applications of diverse types of colloidal hybrid metal–semiconductor nanostructures reported over the last several years. One of the significant challenges in this field is the ability to design and increase the functionality of the single nanostructure. The recent advances in the development of multifunctional materials by interfacing multiple types of materials within a single nanoparticle entity are truly promising but still challenging and have to be further explored. Controlling the properties and the functionalities of nanostructures requires primarily a comprehensible knowledge of the material interfacing mechanism of the sought-after hybrid nanostructure. Thankfully, due to the enormous combinations of diverse hybrid nanomaterials, the scientific community has gained a better insight into the thermodynamic and kinetic parameters, which influence the formation and growth of the hybrid nanomaterials, on the road to develop new multi-component systems with increasing complexity.

Further mechanistic elucidation is expected in the near future with the wide-spreading use of advanced electron microscopy techniques. Liquid-phase *in situ* TEM that allows continuous monitoring has made a significant progress in the elucidation of various metallic NP formation^395–397^ and transformation,^398,399^ as well as other inorganic materials.^400–405^ Other related fields such as superlattice self-assembly from NPs are also treated.^406^ Though some studies dealing with HNSs exist,^407,408^ we envision that a more extensive application of these techniques would thrive, mainly when applied to lower-temperature reactions and some technical limitations (see, for example, a tutorial by De Yoreo and co-workers)^409^ are solved.

Another aspect of characterization is the new information gained using aberration-corrected TEM or STEM: the atomic resolution was already applied to gain mechanistic insight for questions such as SC growth kinetics,^410^ structural evolution by atom diffusion,^411^ surface reconstruction,^185^ composition and bonding in multilayer or multivalent oxides,^412^ crystal structures of growing 1D materials from metallic catalysts (though usually in gas-phase syntheses),^413,414^ defects in 2D materials,^415^ liquid metal/metal-nitride interfaces^416^ and structural changes on the atomic level of SC NRs/M tips.^417^ We expect that its use would expand and allow further understanding of reaction intermediates.

Additionally, advanced analytical microscopy techniques coupled to the microscope will shed light on optical and optoelectronic properties.^418^ Advances in electron energy loss spectroscopy (EELS, used in STEM),^419^ in particular, could give further insight on the connection between composition (*e.g.*, doping levels, oxidation states)^420^ and electronic structure (bandgap, plasmon frequencies, *etc.*^421–423^), temporal transformation of chemical properties^424^ and atomic-resolution of interfaces of complex nanostructures.^425^ Off-axis electron holography could provide detailed electrostatic maps when heterojunctions are formed^426^ or ions are incorporated,^427^ providing priceless information for electronic devices. 3D tomography would allow answering intriguing questions regarding surface structure, as pointed out in a review by Miao and co-workers.^428^

Since intermediate characterization is vital in mechanistic studies, it will not be limited to electron microscopy, and we expect other *in situ* techniques (*e.g.*, X-ray,^429,430^ optical spectroscopy^431–434^) to gain traction alongside the growing use of multimodal imaging.^435^

The integration of hybrid nanostructures in various devices and applications depends tremendously on the progress that is achieved in two directions: simplifying and reducing the synthesis costs and also developing heavy metal-free (abundant and nontoxic elements) and stable hybrid nanostructures; these are yet to emerge.

Much effort is spent to achieve this goal, and we believe that combination of the mechanistic understanding of colloidal nano-synthesis with emphasis on earth-abundant and nontoxic materials^44^ alongside the advances of rational (complex) heterostructure design, discussed in this review, makes using hybrid nanoparticles in a wide range of applications definitely feasible and promising.

## Conflicts of interest

There are no conflicts to declare.

## List of abbreviations

CB: Conduction band
e^−^: Electron
fcc: Face-centered cubic
h^+^: Hole
HAADF: High-angle annular dark field
HER: Hydrogen evolution reaction
HNS: Hybrid nanostructure
ITO: Indium tin oxide
LSPR: Localized surface plasmon resonance
M: Metal
NC: Nanocrystal
NF: Nickel foam
NIR: Near-infrared
NPL: Nanoplate or nanoplatelet
NR: Nanorod
NW: Nanowire
OER: Oxygen evolution reaction
ORR: Oxygen reduction reaction
PCE: Photon-to-electron conversion efficiency
PEC: Photoelectrochemical cell
QD: Quantum dot
rt: Room temperature (25 °C)
SC: Semiconductor
SLS: Solution–liquid–solid
SSP: Single-source precursor
STEM: Scanning transmission electron microscopy
TEM: Transmission electron microscopy
TM: Transition-metal
TMP: Transition-metal phosphide
VB: Valance band
vdW: van der Waals
VLS: Vapor–liquid–solid

### Chemicals and reagents

acac: Acetylacetonate
DDA: Dodecylamine
DDAB: Didodecyldimethylammonium bromide
DDT: Dodecanethiol
DTAB: *n*-Dodecyltrimethylammonium bromide
EtOH: Ethanol
MB: Methylene blue
MeCN: Acetonitrile
MO: Methyl orange
MPA: Mercaptopropionic acid
MUA: Mercaptoundecanoic acid
4-NP: 4-Nitrophenol
OA: Oleic acid
OY: Oleylamine
PEDOT:PSS: Poly(3,4-ethylenedioxythiophene) polystyrene sulfonate
PVP: Polyvinylpyrrolidone
RhB: Rhodamine B
Rh6G: Rhodamine 6G

## Supplementary Material
